# G-protein coupled estrogen receptor 1 contributes to suppression of angiotensin II hypertension via modulation of AMPA GluA1 in the hypothalamic paraventricular nucleus in a mouse model of post-menopause

**DOI:** 10.1186/s13293-026-00922-9

**Published:** 2026-05-23

**Authors:** Teresa A. Milner, Garrett Sommer, Gang Wang, Nour Jaouni, Sumaya Omar Hussein, Michael J. Glass

**Affiliations:** 1 Feil Family Brain and Mind Research Institute, Weill Cornell Medicine, 407 East 61st Street, New York, NY 10065 USA; 2 Weill Cornell Medicine in Qatar, Qatar Foundation, P.O. Box 24144, Education City, Doha, Qatar

**Keywords:** Accelerated ovarian failure, AMPA receptor, Estrogens, Paraventricular nucleus of hypothalamus, Menopause, Neural plasticity

## Abstract

Menopausal hypertension is a leading contributor to adverse health outcomes in women. Although heightened sympathetic activation is implicated in menopausal hypertension, the hypothalamic mechanisms underlying increased blood pressure during ovarian senescence and how this compares to males are not well understood. In this study, treatment with 4-vinylcyclohexene diepoxide (VCD) was used to induce a form of accelerated ovarian failure that parallels the hormonal trajectory of post-menopause (post-AOF). In post-AOF mice, hypertension resulting from 14-day angiotensin II (AngII) infusion was associated with an increase in AMPA GluA1 receptor-mediated, but not NMDA receptor-mediated, currents in sympathoexcitatory neurons in the paraventricular hypothalamic nucleus (PVN). Heightened GluA1 currents in hypertensive post-AOF mice appeared to be mediated by an uncoventional AMPA receptor-AKAP150-associated G-protein coupled estrogen receptor 1 (GPER1) signaling pathway. In male mice, the heightening of GluA1 signaling following hypertension also was dependent on AKAP150, but via the classical protein kinase A signaling pathway. Increased AMPA currents and hypertension were not affected by estrogen receptor beta agonists in post-AOF mice. These results show that both post-AOF and male mice show similar hypertensive responses to slow-pressor AngII but differ in GluA1-GPER1-mediated signaling pathways in the PVN. Moreover, the results in post-AOF mice contrast with prior reports of hypertensive female mice at an early stage of AOF comparable to perimenopause, suggesting that hypertension at early and late ovarian failure are associated with distinct hypothalamic ionotropic glutamate receptor-mediated signaling pathways.

## Highlights

Female mice with advanced accelerated ovarian failure show a hypertensive response to angiotensin II.

Hypertension in mice with advanced ovarian failure involves the non-classical extranuclear G-protein coupled estrogen receptor (GPER1).

Male mice show a hypertensive response comparable to females with ovarian failure, but GPER1 activation does not impact blood pressure.

Signaling pathways underlying hypothalamic neural plasticity supporting hypertension in post-AOF & male mice appear to operate by distinct mechanisms.

## Abstract (plain language summary)

The risk for hypertension increases in women at the time of menopause, but the biological basis for this is unknown. Mechanistic studies of menopausal hypertension have been limited by commonly used animal models of menopause. Here, we employ a model of accelerated ovarian failure (AOF) using 4-vinylcyclohexene diepoxide (VCD) that recapitulates the low estrogen levels seen in women at post-menopause (post-AOF). We found that hypertension in post-AOF mice involves the non-classical extranuclear G-protein coupled estrogen receptor (GPER1) in the paraventricular nucleus of the hypothalamus (PVN), a significant central coordinator of autonomic processes. Although age-matched male mice develop a hypertensive response that is comparable to post-AOF females, they do not respond to GPER1 stimulation. These results indicate that the mechanisms underlying hypertension are distinct in post-AOF and male mice, a finding that may have important implications in understanding sex differences for the neural mechanisms promoting hypertension.

## Introduction

Hypertension is a leading risk factor for cerebral, cardiovascular, renal and other diseases worldwide [1]. Significantly, there are substantial individual differences in hypertension risk, particularly the notable sex divergence in the incidence of hypertension [2, 3]. Indeed, hypertension vulnerability in women varies across the life cycle [4]. Women have a lower susceptibility relative to men at reproductive ages and increasing liability during the menopausal transition (i.e., perimenopause) reaching levels approaching those of males by full menopause (i.e., postmenopause). Despite the increasing recognition of postmenopause as a stage of heightened vulnerability to hypertension, the mechanisms underlying hypertension risk at full menopause and how these mechanisms compare to males are unknown.

Altered ovarian hormones, including irregular cycling and fluctuating levels of estrogen at perimenopause and subsequent loss of estrogen at postmenopause have been associated with elevated blood pressure and sympathetic activity during menopause [5, 6]. Yet, a mechanistic linkage between alterations in estrogen characteristic of natural menopause and hypertension is uncertain, partly because standard animal models (i.e., ovariectomy, aging) lack the temporal pattern of estrogen dynamics seen with human menopause. Ovariectomy is comparable to surgical menopause resulting in the abrupt estrogen depletion [7, 8]. Alternatively, unlike humans, the ovarian senescence of natural aging in rodents result in “estropause”, not complete estrogen depletion, and there is also the further confound of chronological aging which can obscure the actions of hormonal changes [8]. Unlike traditional approaches, 4-vinylcyclohexene diepoxide (VCD) results in accelerated ovarian failure (AOF) that uniquely mimics the course of human menopause [8]. Indeed, VCD-induced AOF initially produces irregular estrogen fluctuations and extended hormone cycles characteristic of human perimenopause (termed “peri-AOF” in rodents) followed by a stage marked by the cessation of estrogen characteristic of postmenopause (“post-AOF”). This approach can be used in the context of rodent hypertension models such as slow-pressor angiotensin II (AngII)-induced hypertension [9]. When administered AngII, cycling female rodents have a lower sensitivity to hypertension compared to males [10], but during VCD-induced AOF mice express hypertension that is comparable to males beginning at peri-AOF and extending into post-AOF [9, 11].

Hypertension during menopause is strongly associated with increased activation of the sympathetic nervous system suggesting that menopausal hypertension is, at least in part, neurogenically mediated [12] In rodents the hypothalamic paraventricular nucleus (PVN) is populated by spinally projecting presympathetic neurons and is thus a key neural component of cardiovascular regulatory circuitry [13, 14]. Our understanding of the PVN’s role in hypertension in females is, however, limited by the lower hypertension sensitivity in cycling female rodents [15, 16], thus much of what is known about hypothalamic contributions to hypertension is based on studies using male rodents.

The activity of PVN sympathoexcitatory neurons during hypertension in males is critically dependent on glutamate-mediated signaling via dendritic NMDA [17, 18] and AMPA [19] receptors. Significantly, coordinated NMDA receptor and estrogen receptor beta (ERß) signaling have been shown to contribute to hypertension at peri-AOF in the VCD model, however, whether a similar signaling pathway is operative during post-AOF is uncertain. Alternatively, AMPA receptors, also are implicated in hypertension in males. However, the functional role of AMPA receptors, including key members of an AMPA receptor-related and estrogen-sensitive signaling complex involving AKAP150 and associated G-protein-coupled estrogen receptor (GPER1), in post-AOF hypertension are unclear. In the present study, the role of hypothalamic AMPA receptor-AKAP150 signaling in hypertension in post-AOF andmale mice was investigated using a combination of molecular, high-resolution imaging, electrophysiological, and gene targeting approaches.

## Materials and methods

All procedures were approved by the Institutional Animal Care and Use Committee of Weill Cornell Medicine and were in accordance with the 2011 Eighth Edition of the National Institute of Health Guide for the Care and Use of Laboratory Animals.

### Animals

Wild-type (WT) C57BL/6J mice (bred in-house) were used in these experiments. Mice were housed in groups of three to four per cage with a 12 h light/12hr dark cycles and *ad libitum* access to food and water. The ser845 (S845) site in GluA1 was studied using a line of mice expressing a serine to alanine substitution at position 845 of this protein [20] (provided by Dr. Richard Huganir). Bacterial artificial chromosome ERβ-enhanced green fluorescent protein (EGFP) reporter mice [21] were originally obtained from Rockefeller University. Mice from these latter two strains were bred in-house and maintained on the C57BL/6 background.

In most studies, female and male mice were littermates or mice were age-matched from the same colony. Assignment of mice to experimental groups was done randomly. Stress was minimized by having the same experimenter conduct experiments at the same time of day throughout the study.

At the end of experiments female mice were anesthetized and estrous phases were determined by vaginal smear cytology [22]. The estrous cycle was classified into three primary phases: (1) proestrus (high estrogen levels; 0.5–1 day long), (2) estrus (declining estrogen levels and increasing progesterone levels; 2–2.5 days long) and (3) diestrus (low estrogen and progesterone levels; 2–2.5 days long). On the day of euthanasia, most females were in estrus or diestrus. Because no estrous cycle-dependent differences were observed in the results, in each experiment female mice from different estrous stages were pooled.

### AOF model of postmenopause

In female mice, AOF was induced by administering VCD [23], which has been shown to model the time course of hormonal changes seen from peri- to post-menopausal phases in humans without the confound of aging (reviewed in [7, 8]). Selective elimination of ovarian primary follicles were achieved with low dose injections of VCD without negatively affecting peripheral tissues, organ weights, liver and kidney function [24, 25, 27] or brain inflammation [24–26].

**Fig. 1:**
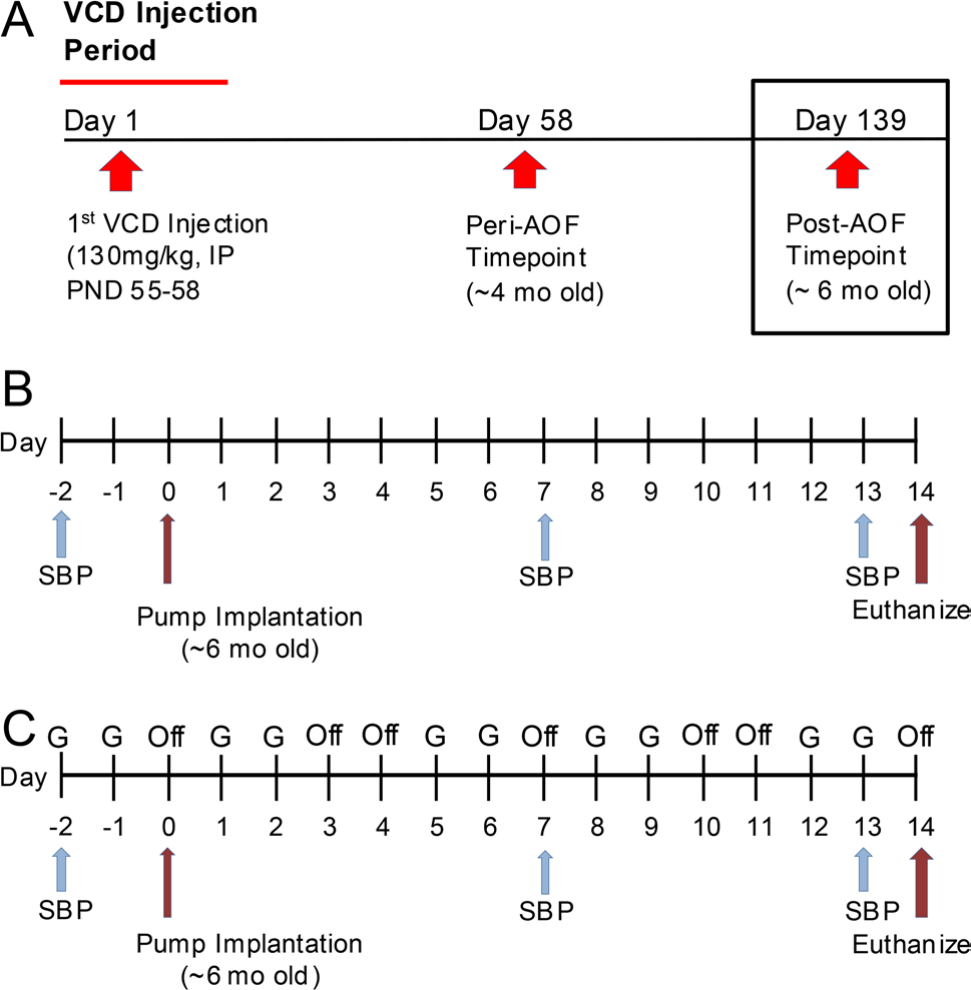
Schematics illustrating experimental procedures. Schematic timeline (**A**) of AOF induction in female mice. Starting between PND 55–58, VCD (130 mg/kg, i.p.) was injected for 3 weeks, 5 days per week. Mice were considered post-AOF starting 139 days following VCD injection, when they are ~ 6 months old. Schematic timeline (**B**) of hypertension induction. Post-AOF female mice and age-matched males were implanted with osmotic minipumps containing AngII (600 ng/kg^− 1^ /min^− 1^) or Sal (saline + 0.1% BSA) for 14 days. SBP was measured via tail-cuff plethysmography for 2 days preceding and 2, 5, 9 and 13 days after minipump implantation, before euthanasia on day 14. Schematic timeline (**C**) of hypertension induction combined with cyclic administration of G-1. Mice were first primed with G-1 (0.1 mg/ml) 2 days before minipump implantation and then injected with G-1 every 2–3 days for a total of 10 injections. G-1 was suspended in Veh (sesame oil)

To induce AOF, gonadally intact female mice (55 to 58-postnatal-day-old) were injected with VCD (130 mg/kg, i.p.; cat. # S453005 Millipore Sigma, St. Louis, MO) in sesame oil (cat. # 8008-74-0 Millipore Sigma) for 5 days per week over a 3-week period [9]. A schematic timeline for the VCD injection procedure is provided in Fig. 1A. As previously characterized [23, 24, 28], at the post-AOF phase (~ 139 days after first VCD injection) mice are about 6 months of age. They are acyclic [23] and have undetectable levels of estrogen, decreased progesterone as well as elevated luteinizing hormone and follicle stimulating hormone and androstenedione levels [7] as seen in postmenopause [8].

### Slow-pressor AngII induction

Prior studies have shown that cycling females are less sensitive to the “slow-pressor” response to AngII when compared to males [9, 28, 29, 31]. However, slow pressor AngII induces hypertension in ovariectomized females [32, 33], aged females [10, 29] and in peri- and post-AOF female mice [9].

A schematic timeline of AngII exposure by osmotic minipumps and systolic blood pressure (SBP) measurement is provided in Fig. 1B. Prior to baseline SBP readings (see below), mice were acclimated to the room and instrumentation for 1 week before minipump implantation. Under isoflurane anesthesia, osmotic minipumps (Alzet, Durect Corp., Cupertino, CA) containing vehicle (saline + 0.1% bovine serum albumin (BSA; cat. # A7030, Millipore Sigma), termed “Sal”) or AngII (600 ng · kg^− 1^ · min^− 1^; cat. # A9525, Millipore Sigma) dissolved in vehicle were implanted subcutaneously. Surgical pump implantation in females occurred ~ 139 days after the first VCD injection (i.e., at post-AOF) and in males that were approximately the same age (about 6 months old). Recording of SBP was made before (baseline), and 7 and 13 days after minipump implantation in awake mice by tail-cuff plethysmography (Model MC4000; Hatteras Instruments, Cary, NC), as described previously [34]. Although the limitations of this method for blood pressure measurement have been discussed previously [10], tail-cuff plethysmography is nonetheless a reliable method for comparing SBP between groups [10, 33, 34, 37] and when applied to slow pressor AngII produces results that are consistent with those using telemetry [17, 38]. Further, since the anatomical methods used here (see below) require aortic arch perfusion fixation of the brain [39], tail-cuff plethysmography is desirable given that it does not compromise the carotid artery, unlike telemetric blood pressure recording [40]. Additionally, tail-cuff methodology does not require single-housing which can increase stress [41]. To control for handling effects, mice were euthanized 1 day after the final SBP measurements. i.e., 14 days after pump implantation [10, 35, 37, 42].

For G-1 administration a cyclical administration schedule was selected since it replicates the pulsatile nature of estrogen levels and may be considered more physiologically relevant than daily hormone exposure [43]. Mice were first primed with a daily dose of G-1 (0.1 mg/ml) two days before minipump implantation and then injected with agonist every 2–3 days for a total of 10 injections. G-1 was suspended in Veh (sesame oil) (Fig. 1C).

### Retrograde labeling of spinally projecting PVN neurons for electrophysiological recording

For microinjection of the retrograde tracer, mice were anesthetized (2–4% isoflurane), and their spinal cords were exposed at the T_2_-T_4_ level through dorsal laminectomy as previously described [37]. Fluorogold (FG 4%; Fluorochrome, Denver CO) was then pressure-ejected (100 nl) bilaterally into the intermediolateral nucleus region of the spinal cord followed by suture of the incision site.

### Whole-cell voltage-clamp

As previously described [37], AMPA and NMDA currents were measured from visually identified FG retrogradely-labeled PVN projection neurons by whole-cell voltage-clamp recording. To determine the roles of AMPA and NMDA currents in AngII-induced hypertension in VCD mice, the whole-cell patch recording was performed in the PVN neurons in brain slices. After anesthesia with 4% isoflurane brains were removed from the cranium and immersed in sucrose-artificial cerebrospinal fluid (s-ACSF) composed of (in mmol/L): 125 sucrose, 26 NaHCO_3_, 5 KCl, 1 NaH_2_PO_4_, 5 MgSO_4_, 1 CaCl_2_, 10 glucose, 4.5 lactic acid, at pH 7.4. Using a Leica VT1000s vibratome, coronal brain slices (200 μm) containing the PVN were cut and collected in s-aCSF. The brain slices were then stored in a custom-designed chamber containing lactic acid-artificial cerebrospinal fluid (l-aCSF) with 95% O_2_ and 5% CO_2_ at 32 °C for 1-hour. The typical l-aCSF was composed of (in mmol/L): 124 NaCl, 26 NaHCO_3_, 5 KCl, 1 NaH_2_PO_4_, 2 MgSO_4_, 2 CaCl_2_, 10 glucose, 4.5 lactic acid, 95% O_2_ and 5% CO_2_, at pH 7.4. The coronal slices containing the PVN were then transferred to the recording chamber and continuously perfused with the typical l-aCSF.

Using the lateral ventricle, fornix, and optic chiasm as landmarks FG-labeled spinal PVN neurons were identified under an E600 epifluorescence microscope (Nikon, Tokyo) using a lucifer yellow filter. The FG-labeled neurons located in the medial one-third of the PVN area between the third ventricle and the fornix were patched for whole-cell voltage-clamp [44] using an Multiclamp 700B amplifier (Molecular Devices, Sunnyvale, CA) at a holding potential of − 60 mV. With respect to NMDA current recording, since NMDA receptor channels are largely blocked by magnesium (Mg^2+^) ions at resting membrane potentials [45], slices were superfused with Mg^2+^-free l-aCSF (in mmol/L 121 NaCl, 5 KCl, 1.8 CaCl_2_, 0.01 glycine, 1 Na-pyruvate, 20 glucose, 26 NaHCO_3_, 1 NaH2PO_4_, 4.5 lactic acid, 95% O_2_ and 5% CO_2_, pH 7.4) to elicit maximal NMDA receptor-mediated inward ionic currents prior to recordings. Voltage-gated sodium channels and non-NMDA receptor channels were blocked with 1 µmol/L of tetrodotoxin (TTX; Millipore Sigma) and 5 µmol/L of 6-cyano-7-nitroquinoxaline-2,3-dione (CNQX; AMPA/Kainate receptor antagonist; Millipore Sigma). Regarding AMPA current recording, the typical l-aCSF was used. Voltage-gated Na^+^ channels and non-AMPA receptor-mediated cation channels were blocked using 1 µmol/L tetrodotoxin (TTX) and 5 µmol/L (2*R*)-amino-5-phosphonovaleric acid (AP5) added to the l-ACSF buffer. Using an ALA-VM4 valve-controlled gravity valve system, control, NMDA (30µmol/L) and AMPA (1 µmol/L) containing buffers were separately perfused toward the patched neuron for 30-seconds using double-barrel microtubing located on the other side of the patch recording electrode [34, 46]. The patch recording pipette tip resistances were 3–5 MΩ as filled with an intracellular solution (in mmol/L): 130 K-gluconate; 10 NaCl, 1.6 MgCl_2_, 1 EGTA, 10 HEPES, 2 Mg-ATP, adjusted to pH 7.3. After formation of a GigaΩ seal, the electrode capacitance was nullified. After breaking the plasma membrane, the cell membrane capacitance (*C*_m_) was read directly from Membrane Test of Window pClamp 11 (Axon Instruments, Union City, CA). Cell membrane capacitance and series resistance were monit1ored throughout the recording, with series resistance generally compensated > 80%. Signals were low-pass filtered at 2 kHz and acquired at a sampling rate of 5–10 kHz. The peak amplitude, rather than the area under the current curve or its decay kinetics, of induced currents were measured.

### Spatiotemporal deletion of GluA1

A neurotropic serotype 2 recombinant adeno-associated virus (AAV) was used to knockdown GluA1. The vector (AAV-GluA1) expressed a GluA1 short hairpin RNA (targeting sequence: 5’-GGAAGCTCTCATTAGCATTAT-3’) and an enhanced green fluorescent protein (GFP) reporter driven by U6 and CMV promoters, respectively (cat #: shAAV-260749; Vector Biolabs, Philadelphia PA). The titer of the rAAV-GluA1 vector was 3.7 × 10^12^ GC/ml. The shRNA was validated for ~ 90% knockdown of mRNA in NIH/3T3 cells. A vector expressing GFP was used as a control (Control vector. cat #: 7004, Vector Biolabs). Bilateral nanoinjections of vector into the PVN were made using previously described stereotaxic surgical procedures [17]. Nanoinjections were made when animals were under 2–4% isoflurane anesthesia. The vectors were injected at a volume of ~ 100 nl/hemisphere at the following coordinates: 0.9 mm posterior and 0.2 mm lateral to bregma, at a depth of 4.8 mm [47]. Nanoinjections were made using a glass pipette (WPI, Sarasota, FL) attached to a Picospritzer II (General Valve Corp., Fairfield, NJ) over a 10-minute interval and the pipette was left in place for an additional 5-10-minutes to prevent leakage. After injections, the mice were allowed to recover in their home cages for 21-days to allow for maximal gene knockdown before testing.

### Light microscopic verification of GFP expression and GluA1 knockdown

After the completion of blood pressure experiments mouse brains were collected for verification of injection sites and assessment of GluA1 and GFP labeling in PVN [17, 18]. Mice were deeply anesthetized with sodium pentobarbital (150 mg/kg, i.p.) and transcardially perfused with 4–5 ml of 1000 units/ml of heparin in 0.9% saline followed by 30 ml of 4% paraformaldehyde (PFA; Electron Microscopy Sciences (EMS), Fort Washington, PA, cat # 19208) in 0.1 M phosphate buffer (PB, pH 7.4). Brains were cut in 5-mm coronal blocks using a brain mold (Activational Systems Inc., Warren, MI). Coronal Sect.  (40 μm thick) through the forebrain were cut using a Vibratome. Sections were stored at − 20 °C in cryoprotectant [39] until immunocytochemical processing.

Sections were rinsed in PB to remove cryoprotectant. Tissue sections were coded with hole punches in the cortex and pooled into containers. Sections were rinsed in 0.1 M Tris-buffered saline (TS; pH 7.6) and then incubated in 0.5% BSA in TS for 30 min. After rinsing in TS, sections were placed in chicken anti-GFP (1:6000) with 0.25% Triton-X100 in 0.1% BSA in TS for 1 day at room temperature (∼23 °C) then 2 days at ∼4 °C. Sections were rinsed in TS followed by an incubation in 1:400 goat anti-chicken IgG (Jackson ImmunoResearch Inc., West Grove, PA) for 30 min. Next, sections were rinsed in TS and then incubated for 30 min in avidin-biotin complex (ABC) at half the manufacturer’s recommended dilution (Vector Laboratories, Burlingame, CA). The bound peroxidase was visualized in the sections by reaction for 6 min in 3,3′-diaminobenzidine (DAB; Sigma-Aldrich Chemical Co., Milwaukee, MI) and hydrogen peroxide.

After immunohistochemical labeling PVN sections were mounted on 1% gelatin coated slides and coverslipped with DPX (cat. # 06522; Millipore Sigma) and visualized using a Nikon Eclipse 80i microscope and photographed equipped with a digital camera (Micropublisher 5.0, Q-imaging, BC, Canada) and IP Lab software (Scanalytics IPLab, RRID: SCR_002775).

The number of GFP labeled cells and GluA1 were assessed on both sides of the PVN in 1–2 mediocaudal section from each group of injected mice. The PVN was traced out Using ImageJ software (RRID: SCR_003070) and the average pixel density within the region was calculated in each hemisphere. Values for each hemisphere were averaged and these values were averaged across animals to obtain group means.

### Light microscopic localization of ERß-GFP

The number of ERß-GFP neurons in the caudal PVN was determined in naïve young adult female (*N* = 5), young adult male (*N* = 5) and post-AOF female ERß-EGFP reporter mice (sections collected in our prior study [48]). Mice were deeply anesthetized with sodium pentobarbital (150 mg/kg, i.p.) and transcardially perfused with 4–5 ml of 1000 units/ml of heparin in 0.9% saline followed by 30 ml 2% PFA and 3.75% acrolein (Polysciences, Washington, PA) in PB (pH 7.4). The brains were removed from the skull and post-fixed for 30 min in 2% acrolein and 2% PFA in PB at room temperature. Coronal Sect.  (40 μm thick) through the PVN were cut using a Vibratome. Sections were stored at − 20 °C in cryoprotectant [39] until immunocytochemical processing. Sections were first washed in PB to remove cryoprotectant and then, to remove excessive aldehydes, incubated in 1% sodium borohydride in PB for 30 min. Sections were then processed for GFP immunohistochemistry as described above.

Prior studies have shown that ERß-GFP neurons correlate with ERß immunolabeled neurons [21]. The number of ERß-GFP labeled cells were counted from one side of the caudal PVN from each mouse. The PVN was traced out Using ImageJ software and the number of ERß-GFP labeled cells was calculated per unit area.

### In situ hybridization

Mice were anesthetized with sodium pentobarbital (150 mg/kg, i.p.) and their brains sequentially fixed by aortic arch perfusion at a flow rate of 20 ml/minute with: 4–5 ml of 1000 units/ml of heparin in 0.9% saline followed by 30 ml of 4% PFA in PB. Brains were extracted from the skull and post-fixed in 4% PFA and 15% sucrose in PB overnight then incubated in 30% sucrose in PB for 1–2 days. Brains were cut into 4–5 mm coronal blocks, frozen with dry ice and stored at -70 °C. Blocks containing the PVN were sectioned on a sliding microtome (30 μm thick) and collected in cryoprotectant solution (30% sucrose and 30% ethylene glycol in PB) before storing at -20 °C.

Expression of mRNA for *Akap5 and Prkaca* in the PVN was assessed using the RNAscope^®^ 2.5 HD Brown Chromogenic Reagent Kit (ACD, Newark, CA) as described previously [49]. For each probe, one section from the mediocaudal PVN (~ 1.0 mm caudal to bregma [47] using prior studies [48, 50] as guides). Prior to mounting, each section was punch coded in the cortex. Cohorts containing sections from each experimental condition were mounted on Superfrost^®^ Plus slides (Cat. # 48311-703 Fischer Scientific) in 1x sterile phosphate buffered saline (PBS) and air dried overnight. The slides were run jointly to ensure that brain sections from each animal were processed under identical conditions.

Prior to processing, slides containing the PVN sections were baked at 60 °C for 1-hour. Next, the sections mounted on slides were incubated in target retrieval solution (Cat # 322000) for 12 min at 40 °C and then treated with Protease III (Cat # 322381) for 30 min at 40 °C. Sections were incubated in *Akap5 and Prkaca* probes (Table 1) at 40 °C for 2-hours. Sections were also processed in tandem with a positive housekeeping control probe *Ppib* and a bacterial negative control probe *DapB*. After probe incubation, the sections underwent six amplification steps according to ACD’s instructions. Tissues then were reacted for 10 min in the chromogen 3, 3’-diaminobenzidine (DAB; ACDBio, Cat # 322310) signal detection step to develop peroxidase staining. Following hybridization, slide-mounted sections were counterstained with 25% Gill’s Hematoxylin No. 1 (Cat. # GHS132; Sigma-Aldrich) followed by dehydration in ascending concentrations of ethanol to xylene. Finally, sections were coverslipped with DPX (Cat # 06522, Sigma-Aldrich).

**Table 1:** In Situ Hybridization Mouse Probes

Probe Name	Gene	Channel	Manufacturer	Catalog #
*Mm-Akap5*	A kinase (PRKA) anchor protein 5	1	ACD	1,258,181-C1
*Mm-Prkaca*	Protein kinase, cAMP dependent, catalytic, alpha	1	ACD	505,581
*Mm-PPIB*	Positive control	1	ACD	313,911
*DapB*	Negative control	1	ACD	310,043

Sections from the mediocaudal PVN were imaged using an Eclipse Nikon 80i microscope (Nikon Inc, Melville, NY). Micrographs of the whole PVN for each cohort were captured at 20x using a Micropublisher 5.0 digital camera (Q-imaging, BC, Canada) and IP Lab software (Scanalytics IPLab, RRID: SCR_002775). Digital images from the whole PVN were analyzed using Halo^®^ Software (Indica Labs, Albuquerque, NM) according to manufacturer’s guidelines (indicalab.com/wp content/uploads/2018/04/MK_51_103_RNAScope_data_analysis_guide_RevB.pdf). This software is capable of distinguishing hematoxylin blue from DAB brown, allowing for the quantification of average probes per unit area in an unbiased manner.

The PVN from one side was traced manually and standard staining color for the probe was entered into the program. After manually setting the parameters for cellular morphology, probe contrast threshold, optical density, and individual probe size, the program automatically analyzed each RNA probe in the whole PVN as well as the periventricular (pv), dorsal paraventricular (dp), and medial parvocellular (mpv) subregions. Probes per unit area in each respective analysis region was determined using Halo^®^ Software. For each mouse the values for each PVN section were averaged and then averaged across animals to provide group means. Collection and analysis of the images were performed by a person blind to the experimental conditions.

### Electron microscopic (EM) immunocytochemical localization of AKAP150

Tissue processing for EM immunohistochemistry was performed using previously described methods [39]. Mice were first deeply anesthetized with sodium pentobarbital (150 mg/kg, i.p.) and their brains fixed by aortic arch perfusion with 3.75% acrolein and 2% PFA in PB. The brains were removed from the skull and post-fixed for 30 min in 2% acrolein and 2% PFA in PB at room temperature. Brains were cut in 5-mm coronal blocks using a brain mold. Coronal brain Sect.  (40 μm) were then cut through the PVN using a Vibratome (VT1000X; Leica Microsystems, Buffalo Grove, IL) and stored at − 20 °C in cryoprotectant (30% sucrose, 30% ethylene glycol in PB) until immunocytochemical processing.

#### Immunocytochemical labeling procedues and tissue processing

Free-floating PVN sections were labeled for AKAP150 using previously described methods [39]. To ensure identical labeling conditions between experimental groups[51], two PVN containing forebrain sections per mouse (0.70–0.94 mm Bregma [52]) were punched for identification, pooled into a single container and then processed in tandem. Sections were first washed in PB to remove cryoprotectant and then, to remove excessive aldehydes, incubated in 1% sodium borohydride in PB for 30 min. Following incubation, sections were rinsed 8–10 times in PB until gaseous bubbles disappeared.

After rinsing in TS sections were blocked with 0.5% BSA in TS for 30 min and rinsed in TS. Sections then were incubated in rabbit anti-AKAP5 (1:100) in 0.1% BSA for 1 day at room temperature (∼23 °C), followed by 2 days at ∼4 °C). Next, sections were rinsed in TS and incubated in donkey anti-rabbit IgG conjugated to 1 nm gold particles (1:50; cat. # 25120 EMS) in 0.01% gelatin and 0.08% BSA in 0.01 M phosphate-buffered-saline (PBS) at 4 °C for 24 h. Sections then were washed in PBS and post-fixed for 10 min in 2% glutaraldehyde in PBS. After PBS washes, sections were placed in 0.2 M sodium citrate buffer (pH 7.4), and bound gold particles were then enhanced for ~ 15 min in a silver solution (SEKL15 Silver enhancement kit, Prod No. 15718 Ted Pella Inc., Redding, CA). All primary and secondary antibody incubations were carried out at 145 rpm, whereas rinses were at 90 rpm on a rotator shaker.

Sections were post-fixed for 1 h in 2% osmium tetroxide in PB, followed by dehydration through a series of alcohols and propylene oxide. Sections were embedded in EMBed 812 (EMS) between two sheets of Aclar plastic. Using a diamond knife (EMS) and a Leica UCT ultratome, ultrathin-sections (~ 70 nm) were cut through the PVN, collected on 400-mesh thin-bar copper grids (T400-Cu, EMS) and counterstained with Uranyless™ (cat. # 22409, EMS) and lead citrate (cat. # 22410, EMS).

Images of analyzed fields from the PVN were obtained using HT7800 transmission electron microscope (Hitachi, Dallas TX) fitted with a digital camera system (v3.2, Advanced Microscopy Techniques, Woburn, MA). AKAP150 appeared as black electron-dense particles [39]. Images of dendrites labeled for AKAP150 were captured at a magnification of 8000x. Dendritic profiles were identified based on standard morphological criteria [51]. These characteristics included a cross-sectional diameter measuring 0.5–2.0 μm and contact by axon terminals which contained numerous small synaptic vesicles. The collection and analysis of micrographs was performed by investigators who were blind to experimental conditions until after the final data analysis and statistics were performed.

To ensure unbiased sampling, 50 randomly selected AKAP150 labeled dendritic profiles were selected from each mouse. Three mice per experimental group were analyzed yielding 150 labeled dendrites per group for the statistical analysis. The location of collected micrographs were indicated on a low magnification photograph of the block surface to ensure that profiles were not double counted. One ultrathin section per block typically yielded the required number of dendrites although images from a non-overlapping region of an additional section were occasionally collected.

Morphological parameters, including perimeter (i.e., plasma membrane), cross-sectional area, average diameter, and major and minor axis lengths for all dendrites were measured using Microcomputer Imaging Device software (MCID Imaging Research Inc., Ont., Canada; RRID: SCR_014278). To normalize SIG counts per unit area the average diameter was determined and used to classify dendrites as large (diameter > 1.0 μm; i.e., proximal) or small (diameter between < 1.0 μm; i.e., distal). The location of AKAP150-SIG particles in each dendritic profile was classified into: (1) On plasmalemma (*On-PM*); (2) Near plasmalemma (*Near-PM*); 3). On and Near plasmalemma (*On + Near PM);* or 4) Cytoplasmic (*Cyto*). Next, the density of AKAP150-SIG particles in dendrites was calculated for: (1) the number of plasma membrane AKAP150-SIG particles on the dendrite perimeter (OnPM/µm); (2) the number of near plasma membrane (< 70 nm) AKAP-SIG particles per perimeter (NearPM/µm); (3) the number of cytoplasmic AKAP150-SIG particles per cross-sectional area (Cyto/µm^2^); and (4) the total number of AKAP150-SIG particles (sum of OnPM, NearPM and Cyto) in a dendritic profile/unit area (Total/µm^2^).

It has been reported that agonist stimulation results in the expected ratio of plasmalemma-to-cytoplasmic SIG labeled receptors [54]. Thus, the presence of plasma membrane receptors identified by SIG labeling corresponds to sites of receptor binding [55]. Given that AKAP150 couples AMPA GluA1 receptors to presumably functional sites on the plasma membrane [56, 57], the subcellular location of AKAP150-SIG particles may provide some functionally important information. It would be expected that the identification of Near plasma membrane SIGs comprises a pool from which receptors can be added or removed from the plasma membrane. SIGs in the cytoplasm represent receptors that are being recycled, stored and/or transported between the soma or another cellular compartment, or degraded [58, 59].

### Antibodies

#### AKAP150

To label AKAP150, a rabbit polyclonal antibody (AKAP5; cat. # bs-6980R; BioSS Antibodies) was used. This antibody was generated against a KLH conjugated synthetic peptide derived from human AKAP5 and specificity has been verified via immunohistochemistry, Western blot, and ELISA (see manufacturer’s datasheet).

#### GFP

To label the gene product of EGFP-expressing transgenic mice, a chicken polyclonal anti-GFP antibody (GFP-1020; RRID: AB_10000240; Aves Lab Inc., San Diego, CA) was used. This antibody was generated against recombinant GFP and specificity has been demonstrated by positive labeling via immunohistochemistry and Western blot in transgenic mice expressing EGFP (see data sheet for EGFP-1020, www.aveslab.com). Additionally, the absence of labeling has been shown in brain sections from mice not expressing EGFP [39, 60].

### Drugs and reagents

AMPA (cat. # A6816), AP5 (cat. # 8054), AngII (cat. # A9525), NMDA (cat. # M3262), TTX (cat. # 554412), and calcium permeable AMPA receptor antagonist 1-Naphthyl acetyl spermine trihydrochloride (Naspm cat. # N193) [61]were obtained from Sigma-Aldrich. GPER1 agonist G-1 (cat # 3677) [62], st-Ht31 (cat # 6286), a cell permeable peptide that inhibits the interaction between AKAP150 and protein kinase A (PKA) [63], and the peptide cell permeable PKA inhibitor PKA 14–22 amide, myristoylated (PKI; cat # 2546) [64]were purchased from Tocris. Also purchased from Tocris were diarylpropionitrile (DPN, cat # 1494), an ERβ “binder/activator” that binds ERβ with high affinity and is a more potent activator of ERβ than ERα, and ERB-041 (prinaberel, cat # 4276), a “binder” as it has a > 200-fold selectivity for ERβ than ERα [65].

### Figure preparation

Micrographs were adjusted to achieve uniformity of brightness, contrast and sharpness using Adobe Photoshop 2020 (Adobe Photoshop, RRID: SCR_014199). Adjusted images were then imported into Microsoft PowerPoint (version 16.16.18 (200112) to create composite figures and perform final adjustments to sharpness, brightness, and contrast. None of the adjustments altered the original content of the raw images. Graphs were generated in Prism 10 (GraphPad Prism, La Jolla, CA; RRID: SCR_002798).

### Experimental design and statistical analysis

#### AMPA and NMDA currents in PVN pre-autonomic neurons

NMDA currents in PVN projection neurons were compared between Sal (*N* = 3 mice; *n* = 4–6 slices, 13–17 neurons) and AngII (*N* = 3 mice; *n* = 4–6 slices, 11 neurons) infused mice in slices from post-AOF females. Additionally, AMPA currents in PVN projection neurons were compared between Sal (*N* = 3 mice; *n* = 4–6 slices, 17 neurons) and AngII (*N* = 3 mice; *n* = 4–6 slices, 14 neurons) infused mice in slices from post-AOF females. In other groups of mice AMPA currents were also compared in post-AOF and male mice. Post-AOF mice were infused with Sal (*N* = 3 mice; *n* = 4–6 slices, 18 neurons) or AngII (*N* = 3 mice; *n* = 4–6 slices, 14 neurons) and male mice were similarly treated with Sal (*N* = 3 mice; *n* = 4–6 slices, 8 neurons) or AngII (*N* = 3 mice; *n* = 4–6 slices, 13 neurons). Unpaired t-tests or One way-ANOVA followed by Tukey’s post-hoc test was used to compare groups. Where non-parametric tests were required the Mann-Whitney U-test was used. In these and subsequent experiments, blood pressure data was analyzed by Repeated Measures (RM) ANOVA or paired t-tests. Data are expressed as means ± SEM in this and all other experiments. Significance was set to an alpha of < 0.05. Here and in subsequent experiments, the statistical analyses were conducted using GraphPad Prism 10 (RRID: SCR_002798).

#### AMPA currents in PVN pre-autonomic neurons after blockade of members of the AKAP150 complex

##### Naspm

To isolate currents from calcium-permeable non-GluA2 *receptors* AMPA currents were measured in PVN-projection neurons in the presence of Naspm. Slices (4–6 /group) were pre-treated with Veh from Sal (*N* = 3; *n* = 17 neurons) or AngII (*N* = 3; *n* = 7 neurons) infused post-AOF mice, or pre-treated with Naspm from Sal (*N* = 3; *n* = 14 neurons) or AngII (*N* = 3; *n* = 14 neurons) infused post-AOF mice.

##### st-Ht31

To assess the role of the AKAP150-PKA binding site, AMPA currents were measured in PVN-projection neurons in the presence of st-Ht31. Slices (3–6 /group) were pre-treated with Veh in Sal (*N* = 3; *n* = 8 neurons) or AngII (*N* = 3; *n* = 17 neurons) infused post-AOF mice or pre-treated with st-Ht31 in Sal (*N* = 3; *n* = 18 neurons) or AngII (*N* = 3; *n* = 20 neurons infused post-AOF mice. Additionally, AMPA currents were also measured in male mice (*N* = 3/group). In Sal-infused mice, slices (4–6/group) were pretreated with Veh (*n* = 4) or st-Ht31 (*n* = 5). In AngII-infused males, slices were pretreated with Veh (*n* = 5) or st-Ht31 (*n* = 5).

##### PKI

To assess the role of PKA, AMPA currents were recorded in slices (3–6/group) after pre-treatment with PKI in Sal (*N* = 3; *n* = 7 neurons) or AngII (*N* = 3; *n* = 7 neurons) infused male mice. To assess the role of PKA, AMPA currents were recorded in slices (3–6/group) after pre-treatment with PKI in Sal (*N* = 3; *n* = 9 neurons) or AngII (*N* = 3; *n* = 8 neurons) infused male mice. In these experiments between-group comparisons were made using unpaired t-tests.

##### Spatial-temporal GluA1 knockdown

To investigate the role of PVN GluA1 on hypertension during ovarian failure, post-AOF mice receiving local injections of control (*N* = 4) or AAV-shRNA (*N* = 6) vectors were infused with AngII. Blood pressure was compared by Two-way RM ANOVA followed by Tukey’s test.

##### Hypertension in S845A mice

To investigate the role of the GluA1 S845 site in hypertension, mutant S845A male mice were infused with AngII (*N* = 11) and were compared to similarly treated WT mice (*N* = 10). WT (*N* = 6) and S845A (*N* = 7) male mice were also treated with Sal. Additionally, post-AOF WT (*N* = 4) and S845A (*N* = 9) mice were infused with AngII. And other WT (*N* = 3) and S845A (*N* = 5) mice were infused with Sal. Blood pressure was compared by Two-way RM ANOVA followed by Tukey’s test separately in male and post-AOF mice.

##### In situ hybridization

Gene expression was measured by in situ hybridization in tissue sections obtained from six groups of mice: (1) Oil vehicle treated female mice given Sal (*N* = 7), (2) Oil female mice given AngII (*N* = 7), (3) Post-AOF mice infused with Sal (*N* = 7), (4) Post-AOF mice treated with AngII (*N* = 7), (5) age-matched males infused with Sal (*N* = 6), or (6) males treated with AngII (*N* = 6). Each experimental group was analyzed for: (1) the number of particles overlying each cell; (2) the number of probe-containing cells; and (3) the pixel density of the particles. For each mouse the values for each PVN section were averaged and then averaged across animals to provide group means. Data were analyzed by unpaired t-tests.

##### EM analysis of AKAP150 distribution in dendrites of PVN neurons

The subcellular distribution of AKAP150 by immunoEM was assessed in six groups of mice: (1) Sal Non-AOF (Oil) females; (2) AngII Non-AOF (Oil) females; (3) Sal post-AOF females; (4) AngII post-AOF females; (5) Sal males; and (6) Ang males (*N* = 3/group). Relative changes in the subcellular distribution of AKAP150 in dendrites was determined. For this, AKAP150 SIG labeling all dendrites and in large and in small dendrites from the experimental groups was determined by specifically designed methods [39]. For this analysis, the distribution of AKAP150 SIG particles in 50 randomly selected dendrites per mouse was determined. The number of SIG particles then was calculated per µm of membrane (on and near PM) or per µm^2^ (cytoplasmic and total) for each dendrite. Inferred in this analysis are corrections for errors related to spatial location, since dendritic profiles within each section are collected from a single plane, as well as differences in dendritic area. Two-way ANOVA with Fisher’s LSD post-hoc tests were used to compare AKAP150 SIG particle density in the dendrites between groups.

##### AMPA currents in PVN projection neurons from hypertensive post-AOF mice during G-1 exposure

To investigate the effect of GPER1 activation on AMPA currents in the PVN of hypertensive post-AOF mice, currents were measured in PVN projection neurons in slices (4–6/group) pre-treated with Veh from Sal (*N* = 3; *n* = 7 neurons) or AngII (*N* = 3; *n* = 9 neurons) infused post-AOF mice or pre-treated with G-1 from Sal (*N* = 3; *n* = 6 neurons) or AngII (*N* = 3; *n* = 10 neurons) infused post-AOF mice. Between-group comparisons were made using One way-ANOVA followed by Tukey’s post-hoc test.

##### Expression of AKAP150 complex member genes in the PVN of hypertensive mice administered G-1

To investigate the effect of GPER1 stimulation on PVN *Akap5*, *Gria1*, and *Prkaca* gene expression 8 groups of mice were tested: (1) post-AOF mice infused with Sal and administered Veh (*N* = 6), (2) post-AOF mice infused with Sal and administered G-1 (*N* = 8), (3) post-AOF mice infused with AngII and administered Veh (*N* = 8), (4) post-AOF mice infused with AngII and given G-1 (*N* = 5), (5) Males infused with Sal and treated with Veh (*N* = 5), (6) males infused with Sal and given G-1 (*N* = 5), (7) males infused with AngII and administered Veh (*N* = 5), and (8) males infused with AngII and treated with G-1 (*N* = 5). For the in situ hybridization analysis, each experimental group was analyzed for: (1) the number of particles overlying each cell; (2) the number of probe-containing cells; and (3) the pixel density of the particles. For each mouse the values for each PVN section were averaged and then averaged across animals to provide group means. Data were analyzed by Three-way ANOVA followed by Fisher’s LSD post-hoc test.

##### Measurement of SBP in AngII-infused mice with GPER agonist administration

The role of GPER1 in hypertension was investigated in 8 groups mice. Treatments included female mice given (1) VCD, Sal and Veh (*N* = 8), (2) VCD, Sal and G-1 (*N* = 6), (3) VCD, AngII, and Veh (*N* = 9), (4) VCD, AngII and G-1 (*N* = 10). Additionally male mice were given (5) Sal and Veh (*N* = 5), (6) Sal and G-1 (*N* = 5), (7) AngII and Veh (*N* = 6), or (8) AngII and G-1 (*N* = 7). Between-group comparisons in blood pressure were made using RM ANOVA followed by Tukey post-hoc test.

##### The role of ERß on AMPA currents and SBP in PVN neurons from hypertensive post-AOF mice

To investigate the effect of ERß activation on AMPA signaling in the PVN of hypertensive post-AOF mice, AMPA currents were measured in PVN projection neurons in slices (4–6/group) pre-treated with Veh from Sal (*N* = 3; *n* = 7 neurons) or AngII (*N* = 3; *n* = 9 neurons) infused post-AOF mice or pre-treated with ERB-041 (10 µmol) or DPN (10 µmol) from Sal (*N* = 3; *n* = 6 neurons) or AngII (*N* = 3; *n* = 10 neurons) infused post-AOF mice. Between-group comparisons were made using One way-ANOVA followed by Tukey’s post-hoc test. In separate groups of mice, SBP was measured in mice infused with AngII and co-administered Veh (*N* = 6) or DPN (1 mg/ml; *N* = 6). Immunolabeling for ERß in the PVN was assessed in male (*N* = 5), non-AOF (*N* = 5) and post-AOF (*N* = 4) mice. Between group comparisons were made by One-way ANOVA followed by Welch’s t-tests.

## Results

### AMPA currents are selectively elevated in spinally-projecting PVN neurons from post-AOF hypertensive mice

In male rodents AngII hypertension involves the activity of glutamatergic AngII receptor-expressing subfornical organ neurons that project to the PVN [66, 67]. Further, PVN neurons projecting to the spinal cord (pre-sympathetic) also show heightened NMDA receptor-mediated signaling during hypertension in males [37]. Elevated NMDA signaling in sympathoexcitatory neurons during AngII hypertension also occurs at an early stage of AOF (i.e., peri-AOF) in female mice [11]. However, it is not known if NMDA receptor signaling is heightened in PVN presympathetic neurons at late AOF (i.e., post-AOF).

**Fig. 2:**
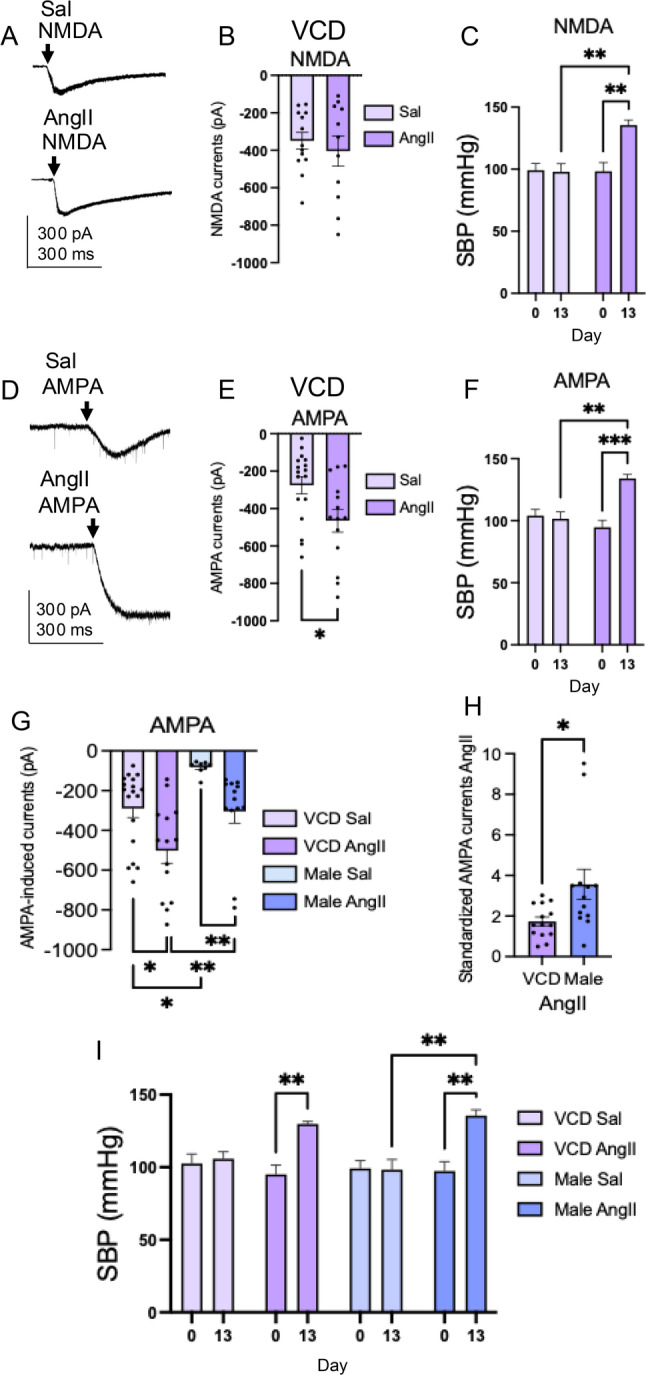
NMDA and AMPA currents in spinally-projecting PVN neurons from post-AOF mice infused with AngII. Current traces (**A**) from PVN projection neurons in slices treated with NMDA from Sal (**top**) and AngII (**bottom**) infused post-AOF mice. Dot plot (**B**) shows NMDA currents in PVN neurons from Sal and AngII-treated mice and histogram (**C**) illustrating blood pressure in these animals. Current traces (**D**) from PVN projection neurons in slices treated with AMPA from Sal (**top**) and AngII (**bottom**) infused post-AOF mice. Dot plot (**E**) showing AMPA currents in PVN projection neurons from Sal and AngII-treated mice and histogram (**F**) illustrating blood pressure in these animals. Dot plots (**G-H**) showing AMPA currents (**G**) in PVN neurons from post-AOF and male mice infused with either Sal or AngII and standardized AMPA currents (**H**; AngII currents/Sal currents) in these neurons. Histograms (**I**) showing SBP in Sal and AngII-infused mice. Data are presented as mean ± standard error of the mean. * *p* < 0.05; ** *p* < 0.01; ***p < 0.001

An assessment of NMDA (30 µM) induced currents in PVN-spinal cord projection neurons of post-AOF mice infused with Sal or AngII was performed [37]. Whole-cell patch recording did not reveal a difference in NMDA-induced currents in PVN-projection neurons from Sal or AngII-infused post-AOF mice (t(22) = 0.6, Unpaired t-test, *p* = 0.537; Fig. 2A-B). However, significant differences in blood pressure were found between treatments (F(1, 4) = 8.6, *p* = 0.042, RM ANOVA; Fig. 2C) with AngII mice compared to Sal showing an increase in SBP from baseline to the final day of recording (*p* < 0.01 Tukey’s test).

There is evidence that AMPA receptor signaling in the PVN plays an important role in blood pressure in male mice but not cycling females [19]. However, it is not known if AMPA currents are impacted in PVN sympathoexcitatory neurons in post-AOF animals. Post-AOF mice were infused with Sal or AngII for 14 days, and whole-cell patch recording was used to measure AMPA currents in PVN-spinal cord projection neurons. In contrast to NMDA, application of AMPA (1 µM) resulted in elevated currents in spinally-projecting PVN neurons in AngII compared to Sal infused post-AOF mice (t(29) = 2.5, Unpaired t-test, *p* = 0.018; Fig. 2D-E). There were significant differences in blood pressure across treatments (F(1, 7) = 25.8, *p* = 0.001, RM ANOVA; Fig. 2F) with AngII mice showing elevated SBP from baseline to the final day of recording (*p* < 0.01, Tukey’s test). These results demonstrate a selective increase in AMPA currents in PVN-projection neurons from post-AOF mice and contrasts with elevated NMDA currents in hypertensive peri-AOF mice, and with prior reports of a heightening of both NMDA and AMPA currents in hypertensive males [17, 19].

Sex differences have been shown with respect to AMPA receptor gene expression [68], synaptic protein distribution [69], and protein interactions [70]. Importantly, in females AMPA receptor signaling is impacted during the estrous cycle [71]. Further, AMPA receptor signaling has also been implicated in hypertension in a sex-dependent manner [19], but it is unclear if AMPA currents are elevated to the same extent in post-AOF and male mice during hypertension in a direct comparison. Post-AOF and age-matched male mice were infused with Sal or AngII (3/group) and AMPA currents were recorded in spinally-projecting PVN neurons. There was a significant effect of treatment (F(3, 49) = 7.9 *p* < 0.001, One-way ANOVA; Fig. 2G) on AMPA currents. Post-hoc testing demonstrated that AMPA currents were higher in PVN-projection neurons from Sal post-AOF compared to Sal male mice (*p* < 0.05, Tukey’s test). Further, currents were significantly higher in PVN-projection neurons from post-AOF compared to male mice when both were infused with AngII (*p* < 0.05, Tukey’s test). Given the significant differences in AMPA currents in Sal-treated mice across the sexes, AMPA currents in Ang-infused animals were standardized by calculating AngII currents as a proportion of Sal currents to assess the relative current change. Despite higher currents in the control condition in post-AOF mice, the relative increase in AMPA currents was found to be greater in AngII-infused males (U = 39, *p* = 0.0104, Mann-Whitney U test, Fig. 2H). With respect to blood pressure, SBP was increased in post-AOF (F(1, 4) = 98.9, *p* = 0.0006; Fig. 2I) and male (F(1, 4) = 13.1, *p* = 0.0221; Fig. 2I) mice, but there was no difference in the magnitude of blood pressure increase between post-AOF and male mice (F(1, 8) = 0.13, *p* = 0.725). These results suggest that post-AOF mice have higher AMPA currents under control conditions, but males have a relatively greater response following AngII hypertension. The basis of this difference, whether a ceiling effect in post-AOF mice, or some other process, is unclear at this time.

### GluA1 currents are heightened in spinally-projecting PVN neurons from post-AOF hypertensive mice

AMPA receptors are heteromers composed of various combinations of GluA1-4 subunits each expressing distinct biophysical properties. Receptors formed by GluA1 and GluA2 subunits are among the most abundant and mediate basal excitatory neurotransmission [72], whereas GluA1 AMPA receptors lacking GluA2 are calcium-permeable and sensitive to Naspm [61]. Further, GluA1 are significantly involved in various forms of neural plasticity [73] and are also implicated in hypertension in a sex-dependent manner [74]. Additionally, GluA1 has been shown to play a role in AngII hypertension in males but not cycling females [19]. However, there is no evidence that GluA1 contributes PVN sympathoexcitatory neurons to in post-AOF AngII-infused animals. Further, signaling at AMPA GluA1 is coordinated by the scaffolding protein AKAP150 which anchors GluA1 and various effector proteins, including PKA, at the plasma membrane [75]. However, there is little evidence that AKAP150 contributes to heightened GluA1-mediated AMPA currents in PVN-projection neurons at post-AOF.

**Fig. 3:**
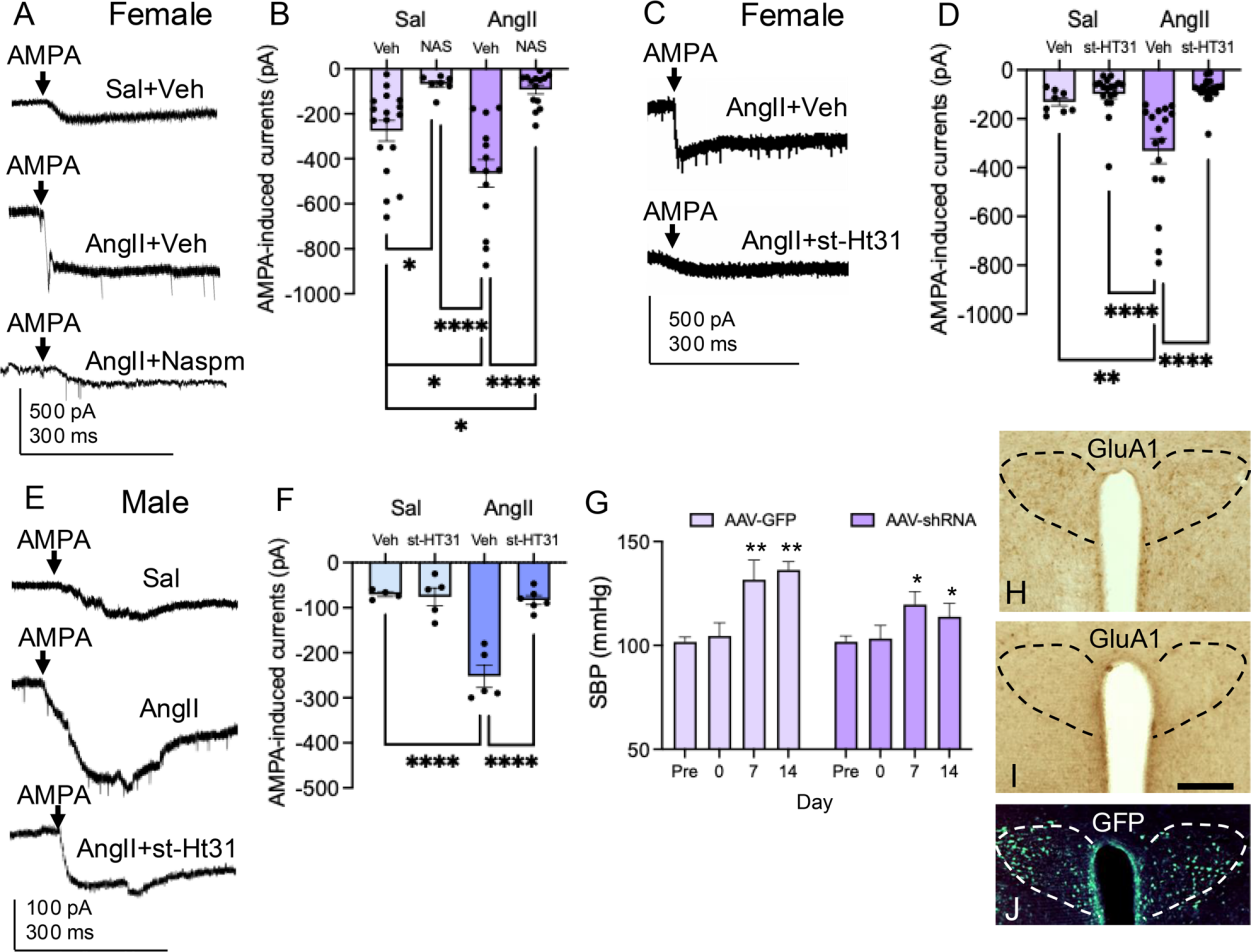
GluA1 currents in spinally projecting PVN neurons from post-AOF mice infused with AngII. Current traces (**A**) from PVN projection neurons in slices treated with AMPA and Veh from Sal (**top**) and AngII (**middle**) infused post-AOF mice as well as slices from AngII-infused post-AOF mice treated with AMPA and Naspm (**bottom**). Dot plot (**B**) showing AMPA currents in PVN projection neurons after pre-treatment with vehicle or Naspm in recordings made from Sal and AngII-treated animals. Current traces (**C**) from PVN projection neurons in slices treated with AMPA and pre-treated with either Veh or st-Ht31 from AngII infused post-AOF mice. Dot plot (**D**) showing AMPA currents in PVN projection neurons after pre-treatment with vehicle or sh-Ht31 in recordings made from Sal and AngII-treated post-AOF animals. Current traces (**E**) from PVN projection neurons in slices treated with AMPA and pre-treated with Veh or st-Ht31 from AngII-infused male mice. Dot plot (**F**) showing AMPA currents in PVN projection neurons after pre-treatment with vehicle or sh-Ht31 in recordings made from Sal and AngII-treated males. Histograms (**G**) showing SBP in rAAV-GluA1shRNA-GFP and control microinjected mice. Light micrographs (**H-J**) illustrating GluA1 immunolabeling of the PVN (area bounded by dashed lines) from AngII-infused post-AOF mice microinjected with rAAV-GluA1shRNA-GFP (**H**; AAV-shRNA) or control vector (**I**; AAV-GFP). Micrograph (**J**) showing GFP immunolabeling in the PVN of a mouse microinjected with rAAV-GluA1shRNA-GFP. Scale bar: 250 μm. Data are presented as mean ± standard error of the mean. * *p* < 0.05; ** *p* < 0.01; **** *p* < 0.0001

Using whole-cell voltage-clamp, AMPA currents were assessed in PVN-spinal cord projection neurons of Sal or AngII treated post-AOF mice. Currents were measured in the presence of the calcium-permeable AMPA receptor blocker Naspm (10 µM) to isolate GluA1 currents [19]. There was a significant effect of treatment on AMPA currents in PVN-projection neurons (F(3, 48) = 14.8, *p* < 0.0001, One-way ANOVA; Fig. 3A-B). It was found that AMPA currents recorded from PVN-projection neurons of AngII-infused compared to Sal-infused mice were significantly reduced when GluA2-lacking AMPA receptors were blocked (*p* < 0.0001, Tukey’s test; Fig. 3). With respect to blood pressure, SBP was increased in AngII-treated mice compared to Sal-treated mice on day 13 of infusion (Sal: Baseline: 100.4 ± 2.6 versus Day 13: 102.3 ± 9.7; AngII: Baseline: 103.9 ± 2.2 versus Day 13: 145.3 ± 8.6; F(1, 4) = 8.4, *p* = 0.044, RM ANOVA).

Functional GluA1-expressing AMPA receptors signal via plasma membrane AKAP150-associated PKA [75], however the role of this signaling complex on AMPA currents during hypertension is not known. In Sal or AngII-treated post-AOF mice, AMPA currents were measured in PVN-spinal cord projection neurons in the presence of st-Ht31 [63] to block the AKAP150-PKA binding site [75]. There was a significant effect of treatment on AMPA currents in PVN-projection neurons (F(3, 59) = 15.0, *p* < 0.0001, One-way ANOVA; Fig. 3C-D). It was found that AMPA currents were reduced in the presence of st-Ht31 compared to vehicle (*p* < 0.001, Tukey’s test). Systolic blood pressure was elevated on day 13 of AngII infusion compared to Sal (Sal: Baseline: 102.9 ± 4.1 vs. Day 13: 108.4 ± 3.1; Ang: Baseline: 100.6 ± 4.4 vs. Day 13: 133.2 ± 7.3; F(1, 5) = 22.9, *p* = 0.005, RM ANOVA).

Male mice were also infused with Sal or AngII and AMPA currents were recorded in spinally-projecting PVN neurons following pretreatment with vehicle or st-HT31. There were significant differences in AMPA currents across treatments (F(3, 16) = 26.6, *p* < 0.0001, One-way ANOVA; Fig. 3E-F). Similar to post-AOF mice, the increase in AMPA currents in neurons from AngII treated vehicle male mice was suppressed following st-Ht31 treatment (*p* < 0.0001, Tukey’s test). Blood pressure was elevated in AngII-infused mice (F(1, 4) = 8.4, *p* = 0.044, RM ANOVA).

Knockdown of PVN GluA1 in male mice has been shown to attenuate elevated blood pressure following slow-pressor AngII infusion [19], however it is not known if GluA1 silencing in the PVN of post-AOF mice influences hypertension. To investigate the functional role of GluA1 on hypertension at advanced ovarian failure, post-AOF mice received bilateral microinjections of an AAV expressing GluA1 shRNA or a control vector. There was a significant effect of vector on blood pressure (F(1, 28) = 4.5, *p* = 0.043, Two-way ANOVA; Fig. 3G-J). In both groups of mice, SBP was elevated at 7 and 14 days post-AngII infusion (*p* < 0.05, Tukey’s test; Fig. 3G), however blood pressure was of a lower magnitude in AAV-GluA1 shRNA mice compared to control vector-treated mice at day 14 (*p* < 0.05, Tukey’s test). GluA1 immunolabeling was reduced in AAV-GluA1 shRNA injected mice (GFP: 109±18.3% pixel density/25mm^2^ vs. GluA1 shRNA: 46±7.9% pixel density 25mm^2^; t(7) = 3.4, *p* < 0.02, Unpaired t-test; Fig. 3. H-J).

### PVN PKA signaling contributes to heightened AMPA currents in hypertensive mice

**Fig. 4:**
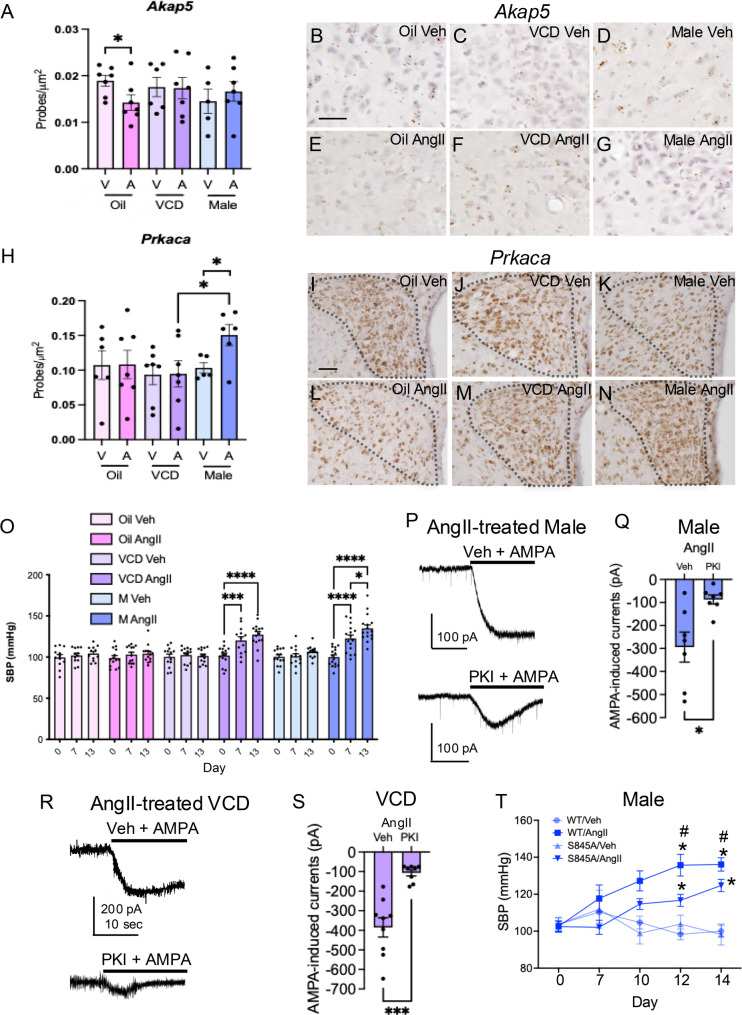
PKA signaling in the PVN of post-AOF and male AngII-infused mice. Dot plot (**A**) showing *Akap5* gene expression in the mpv subregion of the PVN of Oil, VCD and male mice infused with either Sal or AngII. Light micrographs (**B-G**) illustrating *Akap5* mRNA of the PVN (area bounded by dashed lines) from Sal and AngII-infused Oil, VCD and male mice. Dot plot (**H**) showing *Prkaca* gene expression in the PVN of Oil, VCD and male mice infused with either Sal or AngII. Light micrographs (**I-N**) illustrating *Prkaca* mRNA of the PVN (area bounded by dashed lines) from Sal and AngII-infused Oil, VCD and male mice. Histograms (**O**) showing SBP in Sal and AngII-infused mice. Current traces (**P**) showing AMPA currents in PVN projection neurons from AngII-infused mice. Slices were pre-treated with either Veh (**top**) or PKI (**bottom**). Dot plot (**Q**) showing AMPA currents following Veh or PKI pretreatment. Current traces (**R**) showing AMPA currents in PVN projection neurons from AngII-infused post-AOF mice. Slices were pre-treated with either Veh (**top**) or PKI (**bottom**). Dot plot (**S**) showing AMPA currents following Veh or PKI pretreatment. Line graph (**T**) showing SBP in male wild type or S845A mice treated with either Sal or AngII. Scale bar: 10 μm (**B-G**), 50 μm (**I-N**) Data are presented as mean ± standard error of the mean. In A, H, O, Q, S: ^ *p* < 0.07: * *p* < 0.05; *** *p* < 0.001; ****p < 0.0001; In T: *p < 0.05 SBP in WT/AngII and S845A/AngII Days 12 and 14 versus Day 0, # *p* < 0.05 SBP in WT/AngII versus S845A/AngII

Signaling at AKAP150 during hypertension may be related to changes in gene expression, however there is no evidence for this in the PVN of AngII-infused post-AOF mice. Expression of the AKAP150 gene *Akap5* in the PVN following AngII hypertension was assessed by in situ hybridization in Oil-control female, post-AOF, or male mice infused with either Sal or AngII. Blood pressure was elevated in AngII treated post-AOF (Baseline: 102.2 ± 5.6 vs. Day 13: 131.9 ± 5.0) and male (Baseline: 96.1 ± 4.1 vs. Day 13: 140.2 ± 6.4) mice (F(4, 76) = 2.8, *p* = 0.011, RM ANOVA). In control Oil-treated mice, there was a reduction in *Akap5* expression in the PVN of AngII compared to Sal-infused mice (t(12) = 2.3, *p* = 0.041, Unpaired t-test), but not in post-AOF Sal and AngII-infused mice (t(12) = 0.78, *p* = 0.939, Fig. 4A). Additionally male mice infused with Sal or AngII did not differ in PVN *Akap5* gene expression (t(10) = 0.63, *p* = 0.54, Fig. 4A). Representative images for *Akap5* expression in the PVN from the six groups are shown in Fig. 4B-G.

Importantly, PKA is a major effector of the AKAP150 signaling complex critical for heightened GluA1 signaling, which is increased in both male and post-AOF mice [74]. However, little is known about the expression of PKA in post-AOF compared to male mice. The expression of the PKA gene *Prkaca* in the PVN was assessed by in situ hybridization. There was an increase in *Prkaca* mRNA in the PVN of male AngII-infused mice compared to Sal-infused male mice (t(9) = 2.6, *p* = 0.028, Unpaired t-test; Fig. 4H). In contrast, post-AOF AngII and Sal-infused mice did not differ in *Prkaca* expression (t(12) = 0.49, *p* = 0.962; Fig. 4H). Similarly, Sal and AngII-infused Oil-treated female mice did not differ in *Prkaca* expression (t(11) = 0.34, *p* = 0.973 Fig. 4H). Representative images for *Prkaca* expression in the PVN from the six groups are shown in Fig. 4I-N.

The functional role of PKA in AMPA signaling in male mice was also examined by the whole-cell recording in PVN-projection neurons in mice infused with Sal or AngII. Blood pressure was increased in AngII-infused mice (Baseline: 97.9 ± 6.4 vs. Day 13: 135.5 ± 4.0; F(1, 4) = 85.55, *p* = 0.0008, RM ANOVA; Fig. 4O). There was a significant reduction in AMPA currents in PVN neurons pretreated with PKA inhibitor PKA 14–22 amide compared to vehicle in AngII-infused mice (t(7) = 3.2, *p* = 0.019, Welch’s t-test; Fig. 4P, Q). Additionally, AMPA currents after PKA 14–22 were recorded in PVN neurons from post-AOF mice. There was a reduction in AMPA currents following PKA inhibition (t(9.5) = 5.4, *p* = 0.0004, Welch’s t-test; Fig. 4R, S).

Significantly, PKA mediates phosphorylation of GluA1 at the S845 site, that in turn regulates GluA1 plasma membrane localization [74]. However, little is known about the role of the S845 site in blood pressure regulation. To investigate the role of S845 on hypertension, mutant S845A male mice were infused with AngII and were compared to similarly treated WT mice. There was no difference in SBP in male WT and S845A mice treated with Sal (F(2, 33) = 0.35, *p* = 0.708; RM ANOVA). But in AngII-infused males, there was a significant genotype by time interaction (F(2, 57) = 3.3, *p* = 0.043, RM ANOVA; Fig. 4T). Both WT and S845A male mice showed increased SBP over the course of AngII treatment, but S845A mice showed a small but significantly lower reduction compared to WT animals (*p* < 0.05, Holm-Sidak text. Figure 4T). In contrast, post-AOF WT and S845A mice treated with AngII both showed similar increases in SBP (F(2, 22) = 0.19, *p* > 0.98; RM ANOVA; not shown). Additionally, when given Sal WT and S845A mice did not show increased SBP (F(2, 12) = 0.66, *p* = 0.936; RM ANOVA; not shown).

In sum, these results indicate that a pathway involving AKAP150-PKA and GluA1 signaling plays a role in hypertension in male mice.

### AngII hypertension in post-AOF mice results in elevated plasmalemmal AKAP150 in dendrites

Given the increase in AKAP150-dependent GluA1 currents in post-AOF and male hypertensive mice, it might be expected that the subcellular localization of AKAP150, particularly near the plasma membrane where AMPA receptors are functional, would be altered in PVN neurons, as has been shown previously for GluA1 [74]. To investigate the effect of hypertension on AKAP150 localization in PVN neurons, high resolution EM was used to assess the effect of Sal or AngII infusion on the subcellular distribution of AKAP150 in dendrites of PVN neurons of non-AOF, post-AOF mice, and male mice by SIG immunohistochemistry. Further, since distal dendritic processes receive extensive input from glutamatergic axon terminals and play an essential role in excitatory neurotransmission and larger proximal dendrites play critical roles in inhibitory signaling [76], the partitioning ratio of AKAP5 was measured in small (< 1.0 μm diameter) and large (> 1.0 μm) dendritic profiles in the PVN [77].

**Fig. 5:**
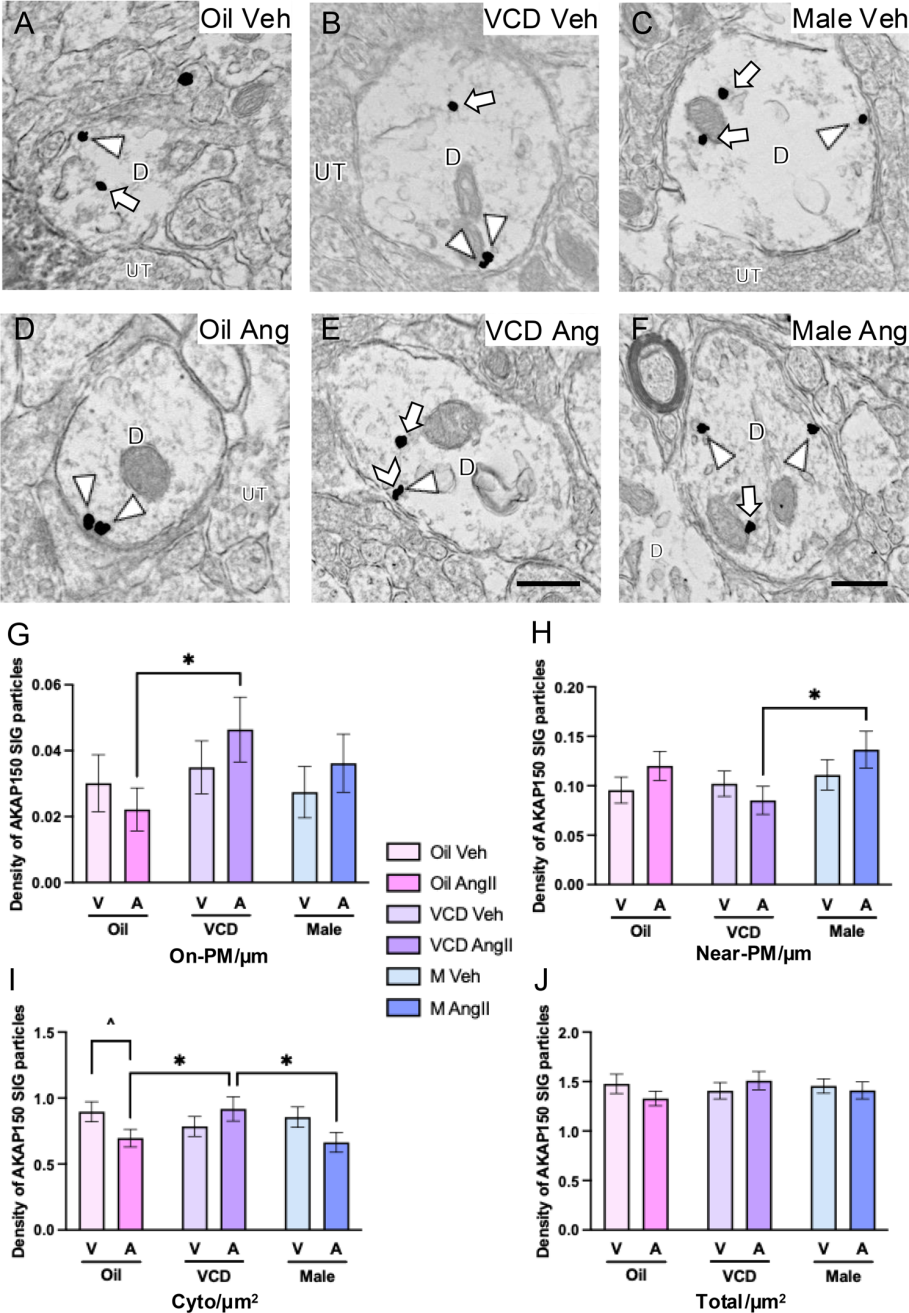
AKAP150 localization in small dendritic profiles in AngII infused mice.Electron micrographs (**A-F**) illustrating immunogold-silver (SIG) labeling for AKAP150 in small dendritic profiles of PVN neurons from Oil, AOF and male mice infused with either Sal or AngII. Arrowheads = on plasma membrane (On-PM), chevrons = near plasma membrane (Near-PM) and arrows = cytoplasm (Cyto). Histograms (**G-J**) showing the density of SIG particles for AKAP150 on the plasma membrane per unit area (On-PM/µm), near the plasma membrane per unit area (Near-PM/µm), in the cytoplasm per unit area (Cyto/µm^2^), and summed across these categories per unit area (Total/µm^2^). Scale bar: 400 nm Data are presented as mean ± standard error of the mean. * *p* < 0.05; ^ *p* < 0.07

Electron micrographs illustrating SIG AKAP150 labeling across conditions are presented in Fig. 5A-F and histograms showing comparisons across subcellular compartments are shown in Fig. 5G-J. A sex/AOF and AngII interaction effect on *Cyto* AKAP150 density was observed in small dendrites (F(2, 753) = 3.071, *p* = 0.047; Two-way ANOVA). Following AngII infusion, there was an increase in the density of *On-PM* AKAP150 (*p* = 0.040; Fig. 5G) and *Cyto* (*p* = 0.044; Fig. 5I), but not *Near-PM* (*p* = 0.093; Fig. 5H) or *On + Near-PM* (*p* = 0.634) AKAP150 in small dendritic profiles in post-AOF mice compared to non-AOF mice. Further, there were no differences in *On-PM* (*p* = 0.67; Fig. 5G), *Near-PM* (*p* = 0.744; Fig. 5H), *On + Near-PM* (*p* = 0.597), or *Cyto* (*p* = 0.291; Fig. 5I) AKAP150 in Sal-infused non-AOF and post-AOF infused mice. In large dendritic profiles, there were no differences in *On-PM* (*p* = 0.6921), *Near-PM* (*p* = 0.883), *On + Near-PM* (*p* = 0.937), or *Cyto* (*p* = 0.766) AKAP150 in AOF mice infused with AngII compared to non-AOF mice. Further, there were no differences in *On-PM* (*p* = 0.816), *Near-PM* (*p* = 0.486) On + *Near-PM* (*p* = 0.461), or *Cyto* (*p* = 0.679) AKAP150 density in Sal-infused Non-AOF and AOF mice.

Males did not differ in *On-PM* (*p* = 0.481), *Near-PM* (*p* = 0.244), *Near + On-PM* (*p* = 0.145) or *Cyto* (*p* = 0.099) AKAP150 in small dendrites across groups. Similarly, there were no differences in *On-PM* (*p* = 0.514), *Near-PM* (*p* = 0.661), *On + Near-PM* (*p* = 0.966), or *Cyto* (*p* = 0.89) AKAP150 in large dendrites. These results indicate that AKAP150 is increased on the plasma membrane of distal sites, known to receive prominent excitatory inputs, of PVN neurons of hypertensive post-AOF mice and are consistent with a previous report of increased plasma membrane GluA1 in post-AOF mice [74].

### Cyclic administration of GPER1 agonist attenuates AngII hypertension in post-AOF mice

The previous electrophysiological, ultrastructural and behavioral results suggest critical sex dependent roles for AKAP150 signaling via GluA1 in hypertension. Hypertension in males was found to be mediated by the canonical pathway involving PKA and GluA1 signaling associated with Ser845. In contrast, hypertension in post-AOF mice was associated with AKAP150 signaling but did not involve the canonical pathway via Ser845. This contrasting pattern of effects in male and post-AOF mice may be mediated by a sex-dependent signaling molecule, such as estrogen, that is associated with AKAP150. Significantly, estrogen exerts a role on AKAP150 signaling through the actions of GPER1 [78, 79], which is expressed in the PVN [48] and is also implicated in experimental hypertension in female rodents [79, 80, 83]. However, the influence of GPER1 activation during post-AOF hypertension is unclear.

**Fig. 6:**
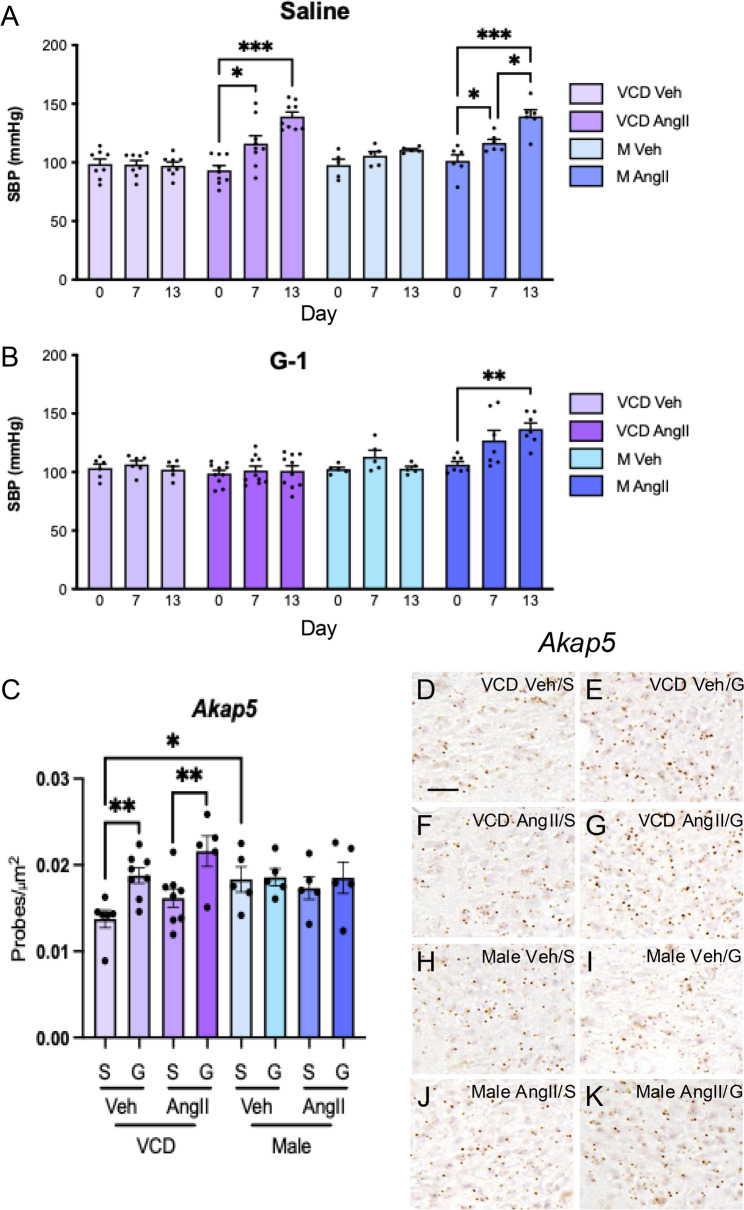
AngII hypertension in post-AOF mice treated with GPER1 agonist. Dot plot (**A**) showing SBP in VCD-treated mice and male mice infused with Veh or AngII and co-administered Sal. Dot plot (**B**) showing SBP in VCD-treated mice and male mice infused with Veh or AngII and co-administered G-1. Dot plots (**C**) showing *Akap5* gene expression in the mpv subregion of the PVN of post-AOF and male mice. Light micrographs (**D-K**) illustrating *Akap5* mRNA expression levels across treatments. Scale bar: 10 μm Data are presented as mean ± standard error of the mean. * *p* < 0.05; ** *p* < 0.01; ***p < 0.001

To investigate the effect of GPER1 agonist administration on hypertension, post-AOF mice were infused with AngII and GPER was stimulated with G-1. There was a significant sex, AngII, and G-1 interaction with respect to SBP (F(2, 110) = 3.5, *p* = 0.027, RM ANOVA). Post-AOF mice infused with Sal and injected with vehicle, did not show an increase in SBP from days 0 to 13. (Fig. 6A) In contrast, post-AOF mice infused with AngII and Veh showed elevated SBP by day 13 (*p* = 0.0005, Tukey’s test; Fig. 6A). However, SBP was unchanged in post-AOF AngII-infused mice treated with G-1 (*p* = 0.9094, Tukey’s test; Fig. 6B). Male mice infused with AngII and injected with saline showed an increase in SBP by day 13 (*p* = 0.0001, Tukey’s test; Fig. 6A). Unlike females, male mice infused with AngII and treated with G-1 had an increase in SBP on day 13 (Fig. 6B). Co-treatment of post-AOF or male mice with Sal and G-1 did not influence blood pressure (Fig. 6A).

To investigate the effect of GPER stimulation on PVN *Akap5* and *Gria1* gene expression, post-AOF mice were infused with Sal and administered either Veh or G-1 and other groups were infused with AngII and administered either Veh or G-1. In addition, males were infused with Sal and treated with Veh or G-1, or infused with AngII and given Veh or G-1. There was a significant effect of G1-administration (F(1, 39) = 10.60, *p* = 0.0023. Three-way ANOVA) and a sex x G1-administration interaction (F(1, 39) = 6.126, *p* = 0.0178, Three-way ANOVA). In post-AOF mice there was an increase in *Akap5* mRNA in animals infused with Sal and co-administered G-1 (*p* = 0.0042, Fisher’s LSD; Fig. 6C, D, E). Similarly, there was an increase in *Akap5* expression in post-AOF mice given AngII and G-1 (*p* = 0.0036, Fisher’s LSD; Fig. 6C, F, G). Males did not differ in *Akap5* mRNA when given G-1 in either Veh (*p* = 0.9055, Fisher’s LSD; Fig. 6C, H, I). or AngII (*p* = 0.5419, Fisher’s LSD; Fig. 6C, J, K) groups. No differences were found across either sex or treatment with respect to *Gria1* expression (not shown).

### Heightened AMPA currents in sympathoexcitatory neurons are suppressed by GPER1 stimulation in post-AOF hypertensive mice

GPER1 is a member of the AKAP150 signaling complex and exerts modulatory actions on GluA1 signaling [79, 83, 84, 87]. Given this background, increased plasma membrane AKAP150 coupled with reduced estrogen in post-AOF mice would be expected to reduce the modulatory actions of GPER1 on GluA1 signaling in PVN-spinal cord projection neurons. In this context it may be hypothesized that stimulating GPER1 would inhibit heightened AMPA currents during hypertension, but there is no evidence for this possibility.

**Fig. 7:**
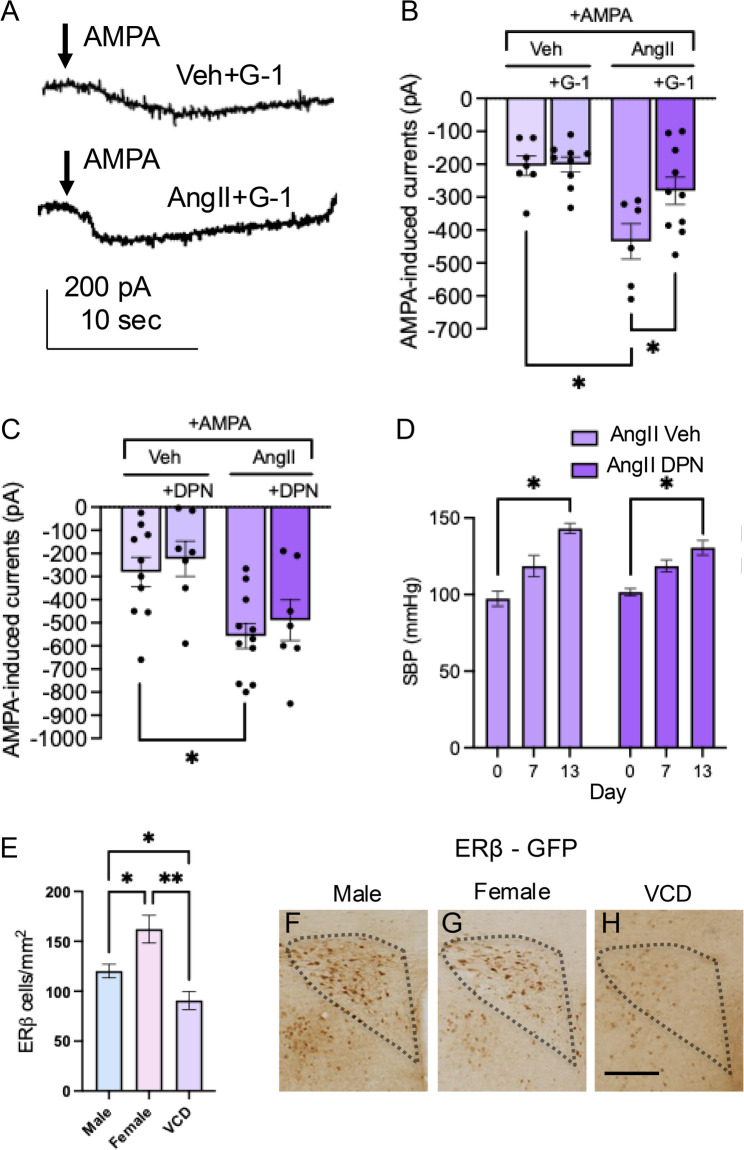
AMPA currents following GPER1 or ERß blockade in PVN projection neurons of post-AOF mice. Current traces (**A**) showing AMPA currents in PVN projection neurons from slices obtained from AngII-infused post-AOF mice following Veh or G-1 pretreatment. Dot plot (**B**) illustrating AMPA currents in PVN projection neurons from slices pre-treated with Veh or AngII and obtained from AngII-infused post-AOF- mice. Dot plot (**C**) showing AMPA currents in PVN neurons from slices treated with Veh or ERß agonists and obtained from post-AOF mice infused with AngII. Histogram (**D**) illustrating SBP in AngII-infused post-AOF mice co-administered Veh or DPN. Histogram (**E**) and light micrographs (**F-H**) showing ERß immunolabeling in the caudal PVN of male and non-AOF or post-AOF females. Scale bar: 100 μm Data are presented as mean ± standard error of the mean. * *p* < 0.05

The role of GPER1 in PVN AMPA signaling was investigated by the whole-cell recording. For this, post-AOF mice were implanted with AngII and currents were recorded in PVN-projection neurons in response to AMPA (30 µM) and vehicle or the GPER1 agonist G-1 (2µM). Other mice were infused with Sal and currents were recorded in the presence of AMPA and vehicle or G-1. There was an effect of treatment on AMPA currents (F(2.02,13.51) = 17.64, *p* = 0.0002, Welch’s ANOVA; Fig. 7A-B). AMPA currents in PVN-projection neurons from AngII-infused mice were higher compared to neurons from Sal-infused mice (*p* < 0.05, Tukey’s test). Significantly, AMPA currents were suppressed in PVN neurons from AngII-infused mice (*p* < 0.05, Tukey’s test) following G-1. These results demonstrate that GPER1 activation in PVN neurons of AngII-infused post-AOF mice inhibits heightened AMPA signaling.

### ERß agonist does not affect AMPA currents in PVN-projection neurons or hypertension in post-AOF mice

There was an effect of treatment on AMPA currents in PVN projection neurons from post-AOF mice (F(3, 31) = 5.6, *p* = 0.0039, One-way ANOVA; 3 mice/group, 7–11 neurons/group; Fig. 7C). There was an increase in AMPA currents in mice infused with AngII and pre-treated with Veh (*p* < 0.02, Tukey’s test; Fig. 7C), but unlike G-1 pre-treatment, there was no reduction when slices were pre-treated with ERß agonists (*p* > 0.8, Tukey’s test; Fig. 7C). The effect of ERß agonist stimulation (Fig. 7D) on blood pressure was also examined in post-AOF mice infused with AngII and treated with Veh (*N* = 6) or agonist DPN (*N* = 6). There was no effect of the ERß agonist on SBP (F(2, 20) = 1.697, *p* = 0.208, treatment by time interaction RM ANOVA; Fig. 7D), unlike what has been reported with ERß stimulation in early AOF (peri-AOF) mice [11]. The number of ERß-GFP cells in the PVN was found to be lower under basal conditions in post-AOF mice compared to non-AOF and male mice (W(2, 6.777) = 8.759, *p* = 0.0132; Welch’s ANOVA; 4–5 mice/group; Fig. 7E-H).

## Discussion

We report for the first time that hypertension induced by slow-pressor AngII is associated with an AKAP150-dependent increase in AMPA GluA1 receptor signaling in presympathetic PVN neurons in post-AOF mice. The increase in GluA1 signaling in post-AOF mice was not accompanied by elevated *Prkaca* gene expression (Fig. 4H), but with an increase in on-plasma membrane AKAP150 in PVN dendrites (Fig. 5G). Further, increased AMPA signaling in post-AOF mice was reversed by activating GPER1 (Fig. 7A-B), an estrogen responsive member of the AKAP150 complex. In male mice the heightening of GluA1 signaling also was dependent on AKAP150 (Fig. 3E-F). In contrast to post-AOF mice, hypertension in male mice was associated with increased *Prkaca* expression (Fig. 4H), but not plasma membrane AKAP150 (Fig. 5G), and was attenuated by interfering with GluA1 Ser845 phosphorylation (Fig. 4T). Additionally, in whole-animal experiments, GPER1 agonist administration inhibited hypertension in post-AOF mice, but not males (Fig. 6B). These results indicate that post-AOF and male mice show similar slow-pressor responses to AngII but differ in AKAP150-GluA1-related signaling pathways in the PVN.

Using the VCD approach, hypertension sensitivity in female mice has been shown to develop at peri-AOF. Further, peri-AOF mice show an increase in NMDA receptor-mediated signaling in ERß PVN neurons [11], which are known to include a high proportion of sympathoexcitatory neurons [21, 48]. The present study showed that contrary to peri-AOF mice, NMDA receptor currents were not elevated in PVN-projection neurons from post-AOF mice (Fig. 2A-B). These results indicate differing roles for the NMDA receptor in PVN sympathoexcitatory neuron adaptability during peri- and post-AOF hypertension.

Glutamate receptors other than the NMDA subtype also have been implicated in hypertension. These include AMPA receptors, which are critical mediators of fast-excitatory signaling and other forms of neural plasticity induced by glutamate [88]. AMPA receptor currents are heightened in PVN-spinal cord projection neurons in male, but not cycling female mice [19]. Like males, the present study revealed an increase in AMPA currents in PVN sympathoexcitatory neurons in post-AOF mice following AngII hypertension (Fig. 2D-E).

Functional AMPA receptors have differing subunit compositions that confer distinct physiological properties [73]. For example, AMPA receptors expressing GluA1, but not GluA2, are calcium permeable, sensitive to Naspm and strongly associated with long-term neural plasticity [72, 89]. Further, they have been implicated in AngII hypertension in male mice [19]. Additionally, GluA1 is part of a plasmalemmal-associated macromolecular complex that includes the scaffolding protein AKAP150, which is expressed in PVN [90] and couples AMPA receptors with PKA [91]. Significantly, members of this GluA1-macromolecular complex are also modulated by estrogen [92] and show sex-dependent actions [93]. There is little evidence regarding the role of GluA1-AKAP150 during hypertension in post-AOF mice [19].

The present study found that the increase in AMPA currents in pre-autonomic PVN neurons from hypertensive post-AOF mice was mediated by the Naspm-sensitive calcium-permeable AMPA receptor (Fig. 3A-B), which is like what has been reported in hypertensive males [19]. Besides the electrophysiological findings, a whole-animal functional study showed that knockdown of PVN GluA1 resulted in an attenuated hypertensive response to AngII in post-AOF mice (Fig. 3G), similar to reports in male mice [19]. Further, we found that AMPA currents in PVN-spinal cord projection neurons of hypertensive post-AOF mice were inhibited by blocking the AKAP150-PKA binding site (Fig. 3C-D), indicating that the heightened calcium-permeable AMPA currents are dependent on the AKAP150-PKA complex. These results in post-AOF mice are similar to the reduction in AMPA currents by blocking the AKAP150-PKA site in hypertensive male mice (Fig. 3E-F).

Given the importance of PKA in AKAP150-GluA1 signaling, expression of the PKA gene *Prkaca* also was measured by in situ hybridization in the PVN of hypertensive post-AOF and male mice. Significantly, expression of the *Prkaca* (Fig. 4H-N), but not *Akap5* (Fig. 4A-G), was increased only in hypertensive male mice. Further, it was shown by electrophysiology that inhibiting PKA attenuated the increase in AMPA currents in PVN-projection neurons from hypertensive male mice (Fig. 4P-Q). PKA has been reported to mediate phosphorylation of GluA1 and plays an important role in protein trafficking to the neuronal plasma membrane [73]. Prior reports point to altered plasma membrane AMPA GluA1 in PVN neurons during hypertension [19, 74]. Plasma membrane GluA1 localization is regulated by phosphorylation of key cytoplasmic residues, particularly at Ser845 by PKA [72, 94]. In the present study, the role of the PKA phosphorylation site Ser845 in hypertension was examined in mice expressing a serine to alanine substitution at this site [20]. We found that S845A mutant male mice showed a small but significant reduction in AngII hypertension (Fig. 4T).

The contrasting patterns of GluA1 signaling in post-AOF and male mice point to a distinction in AKAP150-complex signaling in PVN neurons during hypertension in male and AOF mice that may be dependent on estrogen signaling. Importantly, GPER1 is a non-classical membrane-bound estrogen receptor [94–96, 99] that can also signal through protein-protein interactions to influence glutamate-mediated neural transmission. There is evidence that GPER1 modulates excitatory transmission and neural plasticity via interactions with GluA1-expressing AMPA receptors, which is achieved via GPER1’s participation in a complex with AKAP150 [79, 83, 84, 87]. A significant role for GPER1 in neural plasticity is consistent with GPER1’s association with key postsynaptic density proteins [100], ability to rapidly affect dendritic spine density in a model of learning [101], and ability to modulate neural plasticity in the hippocampus following agonist exposure [102].

Significantly, GPER1 is expressed in the PVN [103], where it is found in somata and dendrites of neurons [48]. Further, the majority of GPER1-expressing PVN neurons co-express ERß, characteristic of PVN-spinal cord projection neurons [10], as shown by dual labeling electron microscopy performed in female mice [48]. However, little is known about the role of PVN GPER1 in AMPA signaling or hypertension in post-AOF mice. By electrophysiological analysis, the present study provided evidence that GPER1 agonist G-1 inhibited the increase in AMPA currents in sympathoexcitatory neurons in hypertensive post-AOF mice (Fig. 7A-B). The combined electrophysiological and high-resolution anatomical data (Fig. 5) suggest that GPER1 is positioned for activation even in a low-estrogen environment, and is capable of responding to agonist by reducing AMPA receptor-mediated excitatory currents in PVN presympathetic neurons.

Evidence from in vivo rodent models also indicates that GPER1 signaling has an important role in hypertension. Specifically, GPER1 expression fluctuates in rodents during changes in hormonal status [103, 104, 106] and during hypertension [107]. Additionally, GPER1 has been shown to play a significant role in blood pressure regulation [82, 83]in an estrogen-dependent manner [107, 108, 111]. Significantly, GPER1 may have translational relevance for hypertension given genomic evidence that a hypofunctional GPER1 gene variant is linked to increased blood pressure only in women [112]. The present study found that cyclic administration of the GPER agonist G-1 during 14-day AngII infusion blocked the increase in blood pressure in post-AOF mice, but not in males or Oil-treated females (Fig. 6B). Interestingly, G-1 administration was also associated with an increase in *Akap5* gene expression in the PVN (Fig. 6C). The increase in *Akap5* suggests that stimulating GPER1 can influence the expression of a molecule that is important for GPER1’s ability to respond to ligand and signal at functional plasma membrane sites. However, this response is likely not due to hypertension, since it occurred in both Sal and AngII-infused post-AOF mice, pointing to a decrease in estrogen as a possible mechanism. This would also be consistent with the finding that Oil-infused mice that would be expected to have normal estrogen levels showed a decrease in *Akap5* when given AngII (Fig. 4A).

The present findings expand on prior studies into the role of glutamate and estrogen receptor signaling in the PVN during hypertension in females. Specifically, we previously reported that young cycling females given AngII do not become hypertensive and show no changes in plasma membrane localization of either NMDA GluN1 or AMPA GluA1 in PVN neurons [9, 74]. In contrast, peri-AOF females show an ERß-reversible increase in blood pressure that is accompanied by elevated plasma membrane GluN1 but not GluA1 [9, 74]. Yet a third pattern emerges during post-AOF. Following AngII post-AOF mice show GPER1, but not ERß-mediated hypertension that is accompanied by increased plasma membrane GluA1 but not GluN1. This evidence indicates that distinct cellular pathways involving select glutamate and estrogen receptors contribute to plasticity in the PVN during AngII-dependent hypertension at early and late stages of ovarian failure.

## Data Availability

The datasets supporting the conclusion of this article are available in the hyperlink to dataset [https://wcm.box.com/s/yv0ftna4q30jeiok40fzah9abpcs2frx](https:/wcm.box.com/s/yv0ftna4q30jeiok40fzah9abpcs2frx).

## Abbreviations

AKAP150: A-kinase anchoring protein 150
AngII: Angiotensin II
AOF: accelerated ovarian failure
GPER1: G-protein coupled estrogen receptor 1
Naspm: 1-Naphthyl acetyl spermine trihydrochloride
PVN: Paraventricular nucleus of the hypothalamus
Sal: Saline
SBP: Systolic blood pressure
SIG: Silver-intensified immunogold
VCD: 4-vinylcyclohexene diepoxide

## References

[1] Zhou B, Perel P, Mensah GA, Ezzati M. Global epidemiology, health burden and effective interventions for elevated blood pressure and hypertension. Nat Rev Cardiol. 2021;28:1–18. 10.1038/s41569-021-00559-8 PMC8162166 34050340

[2] Gerdts E, Sudano I, Brouwers S, Borghi C, Bruno RM, Ceconi C, et al. Sex differences in arterial hypertension. Eur Heart J. 2022;43:4777–88. 36136303 10.1093/eurheartj/ehac470 PMC9726450

[3] Connelly PJ, Currie G, Delles C. Sex Differences in the Prevalence, Outcomes and Management of Hypertension. Curr Hypertens Rep. 2022;24:185–92. 35254589 10.1007/s11906-022-01183-8 PMC9239955

[4] D’Ignazio T, Grand’Maison S, Bérubé L, Forcillo J, Pacheco C. Hypertension across a Woman’s lifespan. Maturitas. 2023;168:84–91. 36549261 10.1016/j.maturitas.2022.11.006

[5] Visniauskas B, Kilanowski-Doroh I, Ogola BO, Mcnally AB, Horton AC, Imulinde Sugi A, et al. Estrogen-mediated mechanisms in hypertension and other cardiovascular diseases. J Hum Hypertens. 2023;37:609–18. 36319856 10.1038/s41371-022-00771-0 PMC10919324

[6] Sabbatini AR, Kararigas G. Estrogen-related mechanisms in sex differences of hypertension and target organ damage. Biol Sex Differ. 2020;11:31. 32487164 10.1186/s13293-020-00306-7 PMC7268741

[7] Van Kempen TA, Milner TA, Waters EM. Accelerated ovarian failure: a novel, chemically induced animal model of menopause. Brain Res. 2011;1379:176–87. 21211517 10.1016/j.brainres.2010.12.064 PMC3078694

[8] Marques-Lopes J, Van Kempen TA, Milner TA. Rodent Models of Ovarian Failure. In: Ram JL, Conn PM, editors. Conn’s Handbook of Models for Human Aging. Academic; 2018. pp. 831–43.

[9] Marques-Lopes J, Tesfaye E, Israilov S, Van Kempen TA, Wang G, Glass MJ et al. Redistribution of NMDA Receptors in Estrogen-Receptor-beta-Containing Paraventricular Hypothalamic Neurons following Slow-Pressor Angiotensin II Hypertension in Female Mice with Accelerated Ovarian Failure. Neuroendocrinology. 2017;104:239 – 56. 10.1159/000446073 PMC5381723 27078860

[10] Marques-Lopes J, Van Kempen T, Waters EM, Pickel VM, Iadecola C, Milner TA. Slow-pressor angiotensin II hypertension and concomitant dendritic NMDA receptor trafficking in estrogen receptor beta-containing neurons of the mouse hypothalamic paraventricular nucleus are sex and age dependent. J Comp Neurol. 2014;522:13:3075–90. 10.1002/cne.23569. 24639345 10.1002/cne.23569 PMC4106979

[11] Milner TA, Contoreggi NH, Yu F, Johnson MA, Wang G, Woods C, et al. Estrogen Receptor β Contributes to Both Hypertension and Hypothalamic Plasticity in a Mouse Model of Peri-Menopause. J Neurosci. 2021;41:245190–205. 10.1523/jneurosci.0164-21.2021. 10.1523/JNEUROSCI.0164-21.2021 PMC8211546 33941651

[12] Akins JD, Washio T, Fu Q. Autonomic control of blood pressure in women: The roles of hypertension and aging. Auton Neurosci. 2025;260:103274. 10.1016/j.autneu.2025.103274. 40188759 10.1016/j.autneu.2025.103274 PMC12276993

[13] Li DP, Pan HL. Glutamatergic Regulation of Hypothalamic Presympathetic Neurons in Hypertension. Curr Hypertens Rep. 2017;19:78. 28929331 10.1007/s11906-017-0776-4

[14] Dampney RA, Horiuchi J, Killinger S, Sheriff MJ, Tan PS, McDowall L. Long-term regulation of arterial blood pressure by hypothalamic nuclei: some critical questions. Clin Exp Pharmacol Physiol. 2005;32:419–25. 15854152 10.1111/j.1440-1681.2005.04205.x

[15] Milner TA, Chen RX, Welington D, Rubin BR, Contoreggi NH, Johnson MA, et al. Angiotensin II differentially affects hippocampal glial inflammatory markers in young adult male and female mice. Learn Mem. 2022;29:9265–73. 10.1101/lm.053507.121. 10.1101/lm.053507.121 PMC9488028 36206386

[16] Sommer G, Rodríguez López C, Hirschkorn A, Calimano G, Marques-Lopes J, Milner TA, et al. Estrogen Receptor Beta Agonist Influences Presynaptic NMDA Receptor Distribution in the Paraventricular Hypothalamic Nucleus Following Hypertension in a Mouse Model of Perimenopause. Biology (Basel). 2024;13:10. 10.3390/biology13100819. 10.3390/biology13100819 PMC11505520 39452127

[17] Glass MJ, Wang G, Coleman CG, Chan J, Ogorodnik E, Van Kempen TA, et al. NMDA Receptor Plasticity in the Hypothalamic Paraventricular Nucleus Contributes to the Elevated Blood Pressure Produced by Angiotensin II. J Neurosci. 2015;35:26:9558–67. 10.1523/jneurosci.2301-14.2015. 26134639 10.1523/JNEUROSCI.2301-14.2015 PMC4571498

[18] Woods C, Marques-Lopes J, Contoreggi NH, Milner TA, Pickel VM, Wang G, et al. Tumor necrosis factor alpha-receptor type 1 activation in the hypothalamic paraventricular nucleus contributes to glutamate signaling and angiotensin II-dependent hypertension. J Neurosci. 2021;41:6:1349–62. 10.1523/jneurosci.2360-19.2020. 33303682 10.1523/JNEUROSCI.2360-19.2020 PMC7888211

[19] Wang G, Woods C, Johnson MA, Milner TA, Glass MJ. Angiotensin II infusion results in both hypertension and increased AMPA GluA1 signaling in hypothalamic paraventricular nucleus of male but not female mice. Neuroscience. 2022;485:129-144. 10.1016/j.neuroscience.2021.12.041 PMC9116447 34999197

[20] Crombag HS, Sutton JM, Takamiya K, Lee HK, Holland PC, Gallagher M, et al. A necessary role for GluR1 serine 831 phosphorylation in appetitive incentive learning. Behav Brain Res. 2008;191:2. 10.1016/j.bbr.2008.03.026. 10.1016/j.bbr.2008.03.026 PMC2478746 18455244

[21] Milner TA, Thompson LI, Wang G, Kievits JA, Martin E, Zhou P, et al. Distribution of estrogen receptor beta containing cells in the brains of bacterial artificial chromosome transgenic mice. Brain Res. 2010;1351:74–96. 10.1016/j.brainres.2010.06.038. 20599828 10.1016/j.brainres.2010.06.038 PMC2926254

[22] Turner CD, Bagnara JT. General Endocrinology. Philadelphia: W.B. Saunders; 1971.

[23] Van Kempen TA, Gorecka J, Gonzalez AD, Soeda F, Milner TA, Waters EM. Characterization of neural estrogen signaling and neurotrophic changes in the accelerated ovarian failure mouse model of menopause. Endocrinology. 2014;155:3610–23. 24926825 10.1210/en.2014-1190 PMC4138565

[24] Haas JR, Christian PJ, Hoyer PB. Effects of impending ovarian failure induced by 4-vinylcyclohexene diepoxide on fertility in C57BL/6 female mice. Comp Med. 2007;57:5. 17974126

[25] Sahambi SK, Visser JA, Themmen AP, Mayer LP, Devine PJ. Correlation of serum anti-Mullerian hormone with accelerated follicle loss following 4-vinylcyclohexene diepoxide-induced follicle loss in mice. Reproductive Toxicol (Elmsford NY). 2008;26:2116–22. Epub 2008 Jul 25. 10.1016/j.reprotox.2008.07.005. 10.1016/j.reprotox.2008.07.005 18706995

[26] Wright LE, Christian PJ, Rivera Z, Van Alstine WG, Funk JL, Bouxsein ML, et al. Comparison of skeletal effects of ovariectomy versus chemically induced ovarian failure in mice. J Bone Min Res. 2008;23:81296–303. 10.1359/jbmr.080309. 10.1359/jbmr.080309 PMC3276352 18348702

[27] Brooks HL, Pollow DP, Hoyer PB. The VCD Mouse Model of Menopause and Perimenopause for the Study of Sex Differences in Cardiovascular Disease and the Metabolic Syndrome. Physiol (Bethesda). 2016;31:4:250–7. 10.1152/physiol.00057.2014. 10.1152/physiol.00057.2014 PMC5504385 27252160

[28] Lohff JC, Christian PJ, Marion SL, Arrandale A, Hoyer PB. Characterization of cyclicity and hormonal profile with impending ovarian failure in a novel chemical-induced mouse model of perimenopause. Comp Med. 2005;55:523–7. 16422148

[29] Xue B, Pamidimukkala J, Hay M. Sex differences in the development of angiotensin II-induced hypertension in conscious mice. Am J Physiol. 2005;288:H2177–84. 10.1152/ajpheart.00969.2004 15626687

[30] Girouard H, Wang G, Gallo EF, Anrather J, Zhou P, Pickel VM, et al. NMDA receptor activation increases free radical production through nitric oxide and NOX2. J Neurosci. 2009;29:2545–52. 19244529 10.1523/JNEUROSCI.0133-09.2009 PMC2669930

[31] Tiwari S, Li L, Riazi S, Halagappa VK, Ecelbarger CM. Sex and age result in differential regulation of the renal thiazide-sensitive NaCl cotransporter and the epithelial sodium channel in angiotensin II-infused mice. Am J Nephrol. 2009;30:554–62. 19844087 10.1159/000252776 PMC2853589

[32] Xue B, Zhang Z, Beltz TG, Johnson RF, Guo F, Hay M, et al. Estrogen receptor-beta in the paraventricular nucleus and rostroventrolateral medulla plays an essential protective role in aldosterone/salt-induced hypertension in female rats. Hypertension. 2013;61:6:1255–62. 10.1161/HYPERTENSIONAHA.111.00903. 23608653 10.1161/HYPERTENSIONAHA.111.00903 PMC3893074

[33] Hay M, Xue B, Johnson AK. Yes! Sex matters: sex, the brain and blood pressure. Curr Hypertens Rep. 2014;16:8. 10.1007/s11906-014-0458-4. 10.1007/s11906-014-0458-4 PMC4081455 24929952

[34] Coleman CG, Wang G, Park L, Anrather J, Delagrammatikas GJ, Chan J, et al. Chronic intermittent hypoxia induces NMDA receptor-dependent plasticity and suppresses nitric oxide signaling in the mouse hypothalamic paraventricular nucleus. J Neurosci. 2010;30:36:12103–12. 10.1523/jneurosci.3367-10.2010. 20826673 10.1523/JNEUROSCI.3367-10.2010 PMC2951676

[35] Marques-Lopes J, Lynch MK, Van Kempen TA, Waters EM, Wang G, Iadecola C, et al. Female protection from slow-pressor effects of angiotensin II involves prevention of ROS production independent of NMDA receptor trafficking in hypothalamic neurons expressing angiotensin 1A receptors. Synapse (New York NY). 2015;69:3148–65. 10.1002/syn.21800. 10.1002/syn.21800 PMC4355104 25559190

[36] Capone C, Faraco G, Anrather J, Zhou P, Iadecola C. Cyclooxygenase 1-derived prostaglandin E2 and EP1 receptors are required for the cerebrovascular dysfunction induced by angiotensin II. Hypertension. 2010;55:4:911–7. 10.1161/HYPERTENSIONAHA.109.145813. 20194308 10.1161/HYPERTENSIONAHA.109.145813 PMC2861995

[37] Wang G, Coleman CG, Chan J, Faraco G, Marques-Lopes J, Milner TA, et al. Angiotensin II slow-pressor hypertension enhances NMDA currents and NOX2-dependent superoxide production in hypothalamic paraventricular neurons. Am J Physiol Regul Integr Comp Physiol. 2013;304(12):R1096–106. 10.1152/ajpregu.00367.2012. 23576605 10.1152/ajpregu.00367.2012 PMC3680791

[38] Capone C, Faraco G, Park L, Cao X, Davisson RL, Iadecola C. The cerebrovascular dysfunction induced by slow pressor doses of angiotensin II precedes the development of hypertension. Am J Physiol Heart Circ Physiol. 2011;300(1):H397–407. 10.1152/ajpheart.00679.2010. 20971763 10.1152/ajpheart.00679.2010 PMC3023263

[39] Milner TA, Waters EM, Robinson DC, Pierce JP. Degenerating processes identified by electron microscpic immunocytochemical methods. In: Manfredi G, Kawamata H, editors. Neurodegeneration, Methods and Protocols. New York: Springer; 2011. pp. 23–59. 10.1007/978-1-61779-328-8_3 21913092

[40] Butz GM, Davisson RL. Long-term telemetric measurement of cardiovascular parameters in awake mice: a physiological genomics tool. Physiol Genom. 2001;5:2:89–97. 10.1152/physiolgenomics.2001.5.2.89. 10.1152/physiolgenomics.2001.5.2.89 11242593

[41] Liu N, Wang Y, An AY, Banker C, Qian YH, O’Donnell JM. Single housing-induced effects on cognitive impairment and depression-like behavior in male and female mice involve neuroplasticity-related signaling. Eur J Neurosci. 2020;52:12694–704. 10.1111/ejn.14565. 10.1111/ejn.14565 31471985

[42] Coleman CG, Wang G, Faraco G, Marques Lopes J, Waters EM, Milner TA, et al. Membrane trafficking of NADPH oxidase p47(phox) in paraventricular hypothalamic neurons parallels local free radical production in angiotensin II slow-pressor hypertension. J Neurosci. 2013;33:10:4308–16. 10.1523/jneurosci.3061-12.2013. 23467347 10.1523/JNEUROSCI.3061-12.2013 PMC3616374

[43] Morrison JH, Brinton RD, Schmidt PJ, Gore AC. Estrogen, menopause, and the aging brain: how basic neuroscience can inform hormone therapy in women. J Neurosci. 2006;26:41:10332–48. 10.1523/jneurosci.3369-06.2006. 17035515 10.1523/JNEUROSCI.3369-06.2006 PMC6674699

[44] Li DP, Chen SR, Pan HL. Nitric oxide inhibits spinally projecting paraventricular neurons through potentiation of presynaptic GABA release. J Neurophysiol. 2002;88:2664–74. 12424302 10.1152/jn.00540.2002

[45] Mayer ML, Westbrook GL, Guthrie PB. Voltage-dependent block by Mg2 + of NMDA responses in spinal cord neurones. Nature. 1984;309:5965:261–3. 10.1038/309261a0. 6325946 10.1038/309261a0

[46] Suh YH, Terashima A, Petralia RS, Wenthold RJ, Isaac JT, Roche KW, et al. A neuronal role for SNAP-23 in postsynaptic glutamate receptor trafficking. Nat Neurosci. 2010;13:338–43. 20118925 10.1038/nn.2488 PMC2861127

[47] Paxinos G, Franklin KB. The mouse brain in stereotaxic coordinates. San Diego, CA: Academic; 2000.

[48] Contoreggi NH, Mazid S, Goldstein LB, Park J, Ovalles AC, Waters EM, et al. Sex and age influence gonadal steroid hormone receptor distributions relative to estrogen receptor β-containing neurons in the mouse hypothalamic paraventricular nucleus. J Comp Neurol. 2021;529:9. 10.1002/cne.25093. 10.1002/cne.25093 PMC8053678 33341960

[49] Sommer G, Omar Hussein S, Milner TA, Glass MJ. Differing patterns of ionotropic glutamate receptor subunit gene expression in paraventricular hypothalamic nucleus subregions following angiotensin II hypertension in male mice and female mice with advanced ovarian failure. Neuroendocrinology. 2026;116:115-138. 10.1159/000549929 PMC12742572 41379749

[50] Biag J, Huang Y, Gou L, Hintiryan H, Askarinam A, Hahn JD, et al. Cyto- and chemoarchitecture of the hypothalamic paraventricular nucleus in the C57BL/6J male mouse: a study of immunostaining and multiple fluorescent tract tracing. J Comp Neurol. 2012;520(1):6–33. 10.1002/cne.22698. 21674499 10.1002/cne.22698 PMC4104804

[51] Pierce JP, Kurucz OS, Milner TA. Morphometry of a peptidergic transmitter system: Dynorphin B-like immunoreactivity in the rat hippocampal mossy fiber pathway before and after seizures. Hippocampus. 1999;9. 10.1002/(SICI)1098-1063(1999)9:3<255::AID-HIPO6>3.0.CO;2-S. :3:255 – 76. 10.1002/(SICI)1098-1063(1999)9:3<255::AID-HIPO6>3.0.CO;2-S 10401641

[52] Hof PR, Young WG, Bloom FE, Belichenko PV, Celio MR. Comparative Cytoarchitectonic Atlas of the C57BL/6 and 129/Sv Mouse Brains. Elsevier; 2000.

[53] Peters A, Palay SL, Webster Hd. The fine structure of the nervous system: neurons and their supporting cells. 3 ed. New York: Oxford University Press; 1991.

[54] Haberstock-Debic H, Wein M, Barrot M, Colago EE, Rahman Z, Neve RL, et al. Morphine acutely regulates opioid receptor trafficking selectively in dendrites of nucleus accumbens neurons. J Neurosci. 2003;23:4324–32. 12764121 10.1523/JNEUROSCI.23-10-04324.2003 PMC6741100

[55] Boudin H, Pelaprat D, Rostene W, Pickel VM, Beaudet A. Correlative ultrastructural distribution of neurotensin receptor proteins and binding sites in the rat substantia nigra. J Neurosci. 1998;18:8473–84. 9763490 10.1523/JNEUROSCI.18-20-08473.1998 PMC6792847

[56] Sanderson JL, Gorski JA, Gibson ES, Lam P, Freund RK, Chick WS, et al. AKAP150-anchored calcineurin regulates synaptic plasticity by limiting synaptic incorporation of Ca2+-permeable AMPA receptors. J Neurosci. 2012;32:43:15036–52. 10.1523/jneurosci.3326-12.2012. 23100425 10.1523/JNEUROSCI.3326-12.2012 PMC3504485

[57] Sanderson JL, Scott JD, Dell’Acqua ML. Control of Homeostatic Synaptic Plasticity by AKAP-Anchored Kinase and Phosphatase Regulation of Ca(2+)-Permeable AMPA Receptors. J Neurosci. 2018;38:11:2863–76. 10.1523/jneurosci.2362-17.2018. 29440558 10.1523/JNEUROSCI.2362-17.2018 PMC5852664

[58] Fernandez-Monreal M, Brown TC, Royo M, Esteban JA. The balance between receptor recycling and trafficking toward lysosomes determines synaptic strength during long-term depression. J Neurosci. 2012;32:38:13200–5. 10.1523/jneurosci.0061-12.2012. 22993436 10.1523/JNEUROSCI.0061-12.2012 PMC6621469

[59] Pierce JP, Kievits J, Graustein B, Speth RC, Iadecola C, Milner TA. Sex differences in the subcellular distribution of AT(1) receptors and nadph oxidase subunits in the dendrites of C1 neurons in the rat rostral ventrolateral medulla. Neuroscience. 2009;163:1329–38. 10.1016/j.neuroscience.2009.06.006. 10.1016/j.neuroscience.2009.06.006 PMC2740659 19501631

[60] Volkmann K, Chen YY, Harris MP, Wullimann MF, Koster RW. The zebrafish cerebellar upper rhombic lip generates tegmental hindbrain nuclei by long-distance migration in an evolutionary conserved manner. J Comp Neurol. 2010;518:2794–817. 20506476 10.1002/cne.22364

[61] Tsubokawa H, Oguro K, Masuzawa T, Nakaima T, Kawai N. Effects of a spider toxin and its analogue on glutamate-activated currents in the hippocampal CA1 neuron after ischemia. J Neurophysiol. 1995;74:1218–25. 10.1152/jn.1995.74.1.218. 10.1152/jn.1995.74.1.218 7472325

[62] Dennis MK, Burai R, Ramesh C, Petrie WK, Alcon SN, Nayak TK, et al. In vivo effects of a GPR30 antagonist. Nat Chem Biol. 2009;5:6:421–7. 10.1038/nchembio.168. 19430488 10.1038/nchembio.168 PMC2864230

[63] Gorshkov K, Mehta S, Ramamurthy S, Ronnett GV, Zhou FQ, Zhang J. AKAP-mediated feedback control of cAMP gradients in developing hippocampal neurons. Nat Chem Biol. 2017;13:4425–31. 10.1038/nchembio.2298. 10.1038/nchembio.2298 PMC5362298 28192412

[64] Hashimotodani Y, Nasrallah K, Jensen KR, Chávez AE, Carrera D, Castillo PE. LTP at Hilar Mossy Cell-Dentate Granule Cell Synapses Modulates Dentate Gyrus Output by Increasing Excitation/Inhibition Balance. Neuron. 2017;95:4928–e433. 10.1016/j.neuron.2017.07.028. 10.1016/j.neuron.2017.07.028 PMC5609819 28817805

[65] Harris HA, Albert LM, Leathurby Y, Malamas MS, Mewshaw RE, Miller CP, et al. Evaluation of an estrogen receptor-beta agonist in animal models of human disease. Endocrinology. 2003;144:10:4241–9. 10.1210/en.2003-0550. 14500559 10.1210/en.2003-0550

[66] Bains JS, Ferguson AV. Paraventricular nucleus neurons projecting to the spinal cord receive excitatory input from the subfornical organ. Am J Physiol. 1995;268(3 Pt 2):R625–33. 10.1152/ajpregu.1995.268.3.R625. 7900904 10.1152/ajpregu.1995.268.3.R625

[67] Llewellyn T, Zheng H, Liu X, Xu B, Patel KP. Median preoptic nucleus and subfornical organ drive renal sympathetic nerve activity via a glutamatergic mechanism within the paraventricular nucleus. Am J Physiol Regul Integr Comp Physiol. 2012;302(4):R424–32. 10.1152/ajpregu.00403.2011. 22160544 10.1152/ajpregu.00403.2011 PMC3293509

[68] Achzet LM, Jackson DA. Sex-Dependent Differences in the Ischemia/Reperfusion-Induced Expression of AMPA Receptors. Int J Mol Sci. 2024;25:4. 10.3390/ijms25042231. 10.3390/ijms25042231 PMC10889403 38396906

[69] Knouse MC, McGrath AG, Deutschmann AU, Rich MT, Zallar LJ, Rajadhyaksha AM, et al. Sex differences in the medial prefrontal cortical glutamate system. Biol Sex Differ. 2022;13:166. 10.1186/s13293-022-00468-6. 10.1186/s13293-022-00468-6 PMC9641904 36348414

[70] Wickens MM, Kirkland JM, Knouse MC, McGrath AG, Briand LA. Sex-specific role for prefrontal cortical protein interacting with C kinase 1 in cue-induced cocaine seeking. Addict Biol. 2021;26:5e13051. 10.1111/adb.13051. 10.1111/adb.13051 PMC8865577 34110073

[71] Bechard AR, Hamor PU, Schwendt M, Knackstedt LA. The effects of ceftriaxone on cue-primed reinstatement of cocaine-seeking in male and female rats: estrous cycle effects on behavior and protein expression in the nucleus accumbens. Psychopharmacology. 2018;235:3837–48. 10.1007/s00213-017-4802-7. 10.1007/s00213-017-4802-7 PMC5893281 29197981

[72] Buonarati OR, Hammes EA, Watson JF, Greger IH, Hell JW. Mechanisms of postsynaptic localization of AMPA-type glutamate receptors and their regulation during long-term potentiation. Sci Signal. 2019;12(562):eaar6889. 10.1126/scisignal.aar6889 PMC7175813 30600260

[73] Diering GH, Huganir RL. The AMPA receptor code of synaptic plasticity. Neuron. 2018;100:314–29. 30359599 10.1016/j.neuron.2018.10.018 PMC6214363

[74] Ovalles AC, Contoreggi NH, Marques-Lopes J, Van Kempen TA, Iadecola C, Waters EM, et al. Plasma Membrane Affiliated AMPA GluA1 in Estrogen Receptor beta-containing Paraventricular Hypothalamic Neurons Increases Following Hypertension in a Mouse Model of Post-menopause. Neuroscience. 2019;423:192–205. 10.1016/j.neuroscience.2019.09.026. 31682817 10.1016/j.neuroscience.2019.09.026 PMC7877340

[75] Diering GH, Gustina AS, Huganir RL. PKA-GluA1 coupling via AKAP5 controls AMPA receptor phosphorylation and cell-surface targeting during bidirectional homeostatic plasticity. Neuron. 2014;84:4:790–805. 10.1016/j.neuron.2014.09.024. 25451194 10.1016/j.neuron.2014.09.024 PMC4254581

[76] London M, Hausser M. Dendritic computation. Annual Rev Neurosci. 2005;28:503–32. 16033324 10.1146/annurev.neuro.28.061604.135703

[77] Beckerman MA, Ogorodnik E, Glass MJ. Acute morphine associated alterations in the subcellular location of the AMPA-GluR1 receptor subunit in dendrites of neurons in the mouse central nucleus of the amygdala: Comparisons and contrasts with other glutamate receptor subunits. Synapse. 2013;67:692–704. 23564315 10.1002/syn.21673 PMC4061138

[78] Koitmäe A, Karsten Y, Li X, Morellini F, Rune GM, Bender RA. GPER1 deficiency causes sex-specific dysregulation of hippocampal plasticity and cognitive function. J Endocrinol. 2023;258:3. 10.1530/joe-22-0204. 10.1530/JOE-22-0204 37399525

[79] Clements L, Alexander A, Hamilton K, Irving A, Harvey J. G-protein coupled estrogen receptor (GPER1) activation promotes synaptic insertion of AMPA receptors and induction of chemical LTP at hippocampal temporoammonic-CA1 synapses. Mol Brain. 2023;16:116. 10.1186/s13041-023-01003-3. 10.1186/s13041-023-01003-3 PMC9883958 36709268

[80] Meyer MR, Prossnitz ER, Barton M. GPER/GPR30 and Regulation of Vascular Tone and Blood Pressure. Immunol Endocr Metab Agents Med Chem. 2011;11:255–61. 24999376 10.2174/1871522211108040255 PMC4079007

[81] Meyer MR, Fredette NC, Daniel C, Sharma G, Amann K, Arterburn JB et al. Obligatory role for GPER in cardiovascular aging and disease. Sci Signal. 2016;9(452):ra105. 10.1126/scisignal.aag0240 PMC5124501 27803283

[82] Haas E, Bhattacharya I, Brailoiu E, Damjanović M, Brailoiu GC, Gao X, et al. Regulatory role of G protein-coupled estrogen receptor for vascular function and obesity. Circ Res. 2009;104:3288–91. 10.1161/circresaha.108.190892. 10.1161/CIRCRESAHA.108.190892 PMC2782532 19179659

[83] Mårtensson UE, Salehi SA, Windahl S, Gomez MF, Swärd K, Daszkiewicz-Nilsson J, et al. Deletion of the G protein-coupled receptor 30 impairs glucose tolerance, reduces bone growth, increases blood pressure, and eliminates estradiol-stimulated insulin release in female mice. Endocrinology. 2009;150:2687–98. 10.1210/en.2008-0623. 10.1210/en.2008-0623 18845638

[84] Godó S, Barabás K, Lengyel F, Ernszt D, Kovács T, Kecskés M, et al. Single-Molecule Imaging Reveals Rapid Estradiol Action on the Surface Movement of AMPA Receptors in Live Neurons. Front Cell Dev Biol. 2021;9:708715. 10.3389/fcell.2021.708715. 34631701 10.3389/fcell.2021.708715 PMC8495425

[85] Tutzauer J, Serafin DS, Schmidt T, Olde B, Caron KM, Leeb-Lundberg LMF. G protein-coupled estrogen receptor (GPER)/GPR30 forms a complex with the β(1)-adrenergic receptor, a membrane-associated guanylate kinase (MAGUK) scaffold protein, and protein kinase A anchoring protein (AKAP) 5 in MCF7 breast cancer cells. Arch Biochem Biophys. 2024;752:109882. 10.1016/j.abb.2024.109882. 38211639 10.1016/j.abb.2024.109882 PMC11481754

[86] Broselid S, Berg KA, Chavera TA, Kahn R, Clarke WP, Olde B, et al. G protein-coupled receptor 30 (GPR30) forms a plasma membrane complex with membrane-associated guanylate kinases (MAGUKs) and protein kinase A-anchoring protein 5 (AKAP5) that constitutively inhibits cAMP production. J Biol Chem. 2014;289:32:22117–27. 10.1074/jbc.M114.566893. 24962572 10.1074/jbc.M114.566893 PMC4139225

[87] Gonzalez de Valdivia E, Broselid S, Kahn R, Olde B, Leeb-Lundberg LMF. G protein-coupled estrogen receptor 1 (GPER1)/GPR30 increases ERK1/2 activity through PDZ motif-dependent and -independent mechanisms. J Biol Chem. 2017;292:24:9932–43. 10.1074/jbc.M116.765875. 28450397 10.1074/jbc.M116.765875 PMC5473245

[88] Sheng M, Kim MJ. Postsynaptic signaling and plasticity mechanisms. Science. 2002;298:5594. 10.1126/science.1075333. 10.1126/science.1075333 12399578

[89] Eyigor O, Centers A, Jennes L. Distribution of ionotropic glutamate receptor subunit mRNAs in the rat hypothalamus. J Comp Neurol. 2001;434:1101–24. 10.1002/cne.1167 11329132

[90] Ostroveanu A, Van der Zee EA, Dolga AM, Luiten PG, Eisel UL, Nijholt IM. A-kinase anchoring protein 150 in the mouse brain is concentrated in areas involved in learning and memory. Brain Res. 2007;1145:97–107. 10.1016/j.brainres.2007.01.117. 17321504 10.1016/j.brainres.2007.01.117

[91] Weisenhaus M, Allen ML, Yang L, Lu Y, Nichols CB, Su T, et al. Mutations in AKAP5 disrupt dendritic signaling complexes and lead to electrophysiological and behavioral phenotypes in mice. PLoS ONE. 2010;5:4e10325. 10.1371/journal.pone.0010325. 10.1371/journal.pone.0010325 PMC2859064 20428246

[92] Akama KT, McEwen BS. Estrogen stimulates postsynaptic density-95 rapid protein synthesis via the Akt/protein kinase B pathway. J neuroscience: official J Soc Neurosci. 2003;23:6. 10.1523/JNEUROSCI.23-06-02333.2003 PMC6742036 12657692

[93] Mastro TL, Preza A, Basu S, Chattarji S, Till SM, Kind PC, et al. A sex difference in the response of the rodent postsynaptic density to synGAP haploinsufficiency. Elife. 2020;9. 10.7554/eLife.52656. 10.7554/eLife.52656 PMC6994236 31939740

[94] Liu F, Day M, Muniz LC, Bitran D, Arias R, Revilla-Sanchez R, et al. Activation of estrogen receptor-beta regulates hippocampal synaptic plasticity and improves memory. Nat Neurosci. 2008;11:3334–43. 10.1038/nn2057. 10.1038/nn2057 18297067

[95] Revankar CM, Cimino DF, Sklar LA, Arterburn JB, Prossnitz ER. A transmembrane intracellular estrogen receptor mediates rapid cell signaling. Science. 2005;307:1625–30. 15705806 10.1126/science.1106943

[96] Thomas P, Pang Y, Filardo EJ, Dong J. Identity of an estrogen membrane receptor coupled to a G protein in human breast cancer cells. Endocrinology. 2005;146:624–32. 15539556 10.1210/en.2004-1064

[97] Cheng SB, Graeber CT, Quinn JA, Filardo EJ. Retrograde transport of the transmembrane estrogen receptor, G-protein-coupled-receptor-30 (GPR30/GPER) from the plasma membrane towards the nucleus. Steroids. 2011;76:892–6. 21354433 10.1016/j.steroids.2011.02.018

[98] Romano SN, Gorelick DA. Crosstalk between nuclear and G protein-coupled estrogen receptors. Gen Comp Endocrinol. 2018;261:190–7. 28450143 10.1016/j.ygcen.2017.04.013 PMC5656538

[99] Prossnitz ER, Barton M. Estrogen biology: new insights into GPER function and clinical opportunities. Mol Cell Endocrinol. 2014;389:71–83. 24530924 10.1016/j.mce.2014.02.002 PMC4040308

[100] Waters EM, Thompson LI, Patel P, Gonzales AD, Ye HZ, Filardo EJ, et al. G-protein-coupled estrogen receptor 1 is anatomically positioned to modulate synaptic plasticity in the mouse hippocampus. J Neurosci. 2015;35:2384–97. 25673833 10.1523/JNEUROSCI.1298-14.2015 PMC4323523

[101] Gabor C, Lymer J, Phan A, Choleris E. Rapid effects of the G-protein coupled oestrogen receptor (GPER) on learning and dorsal hippocampus dendritic spines in female mice. Physiol Behav. 2015;149:53–60. 26003497 10.1016/j.physbeh.2015.05.017

[102] Briz V, Liu Y, Zhu G, Bi X, Baudry M. A novel form of synaptic plasticity in field CA3 of hippocampus requires GPER1 activation and BDNF release. J Cell Biol. 2015;210:1225–37. 26391661 10.1083/jcb.201504092 PMC4586750

[103] Xu H, Qin S, Carrasco GA, Dai Y, Filardo EJ, Prossnitz ER, et al. Extra-nuclear estrogen receptor GPR30 regulates serotonin function in rat hypothalamus. Neuroscience. 2009;158:1599–607. 19095043 10.1016/j.neuroscience.2008.11.028 PMC2747636

[104] Spary EJ, Chapman SE, Sinfield JK, Maqbool A, Kaye J, Batten TF. Novel G protein-coupled oestrogen receptor GPR30 shows changes in mRNA expression in the rat brain over the oestrous cycle. Neurosignals. 2013;21:14–27. 22378360 10.1159/000333296

[105] Llorente R, Marraudino M, Carrillo B, Bonaldo B, Simon-Areces J, Abellanas-Pérez P, et al. G Protein-Coupled Estrogen Receptor Immunoreactivity Fluctuates During the Estrous Cycle and Show Sex Differences in the Amygdala and Dorsal Hippocampus. Front Endocrinol (Lausanne). 2020;11:537. 10.3389/fendo.2020.00537. 32849310 10.3389/fendo.2020.00537 PMC7426398

[106] Marraudino M, Carrillo B, Bonaldo B, Llorente R, Campioli E, Garate I, et al. G Protein-Coupled Estrogen Receptor Immunoreactivity in the Rat Hypothalamus Is Widely Distributed in Neurons, Astrocytes, and Oligodendrocytes, Fluctuates during the Estrous Cycle, and Is Sexually Dimorphic. Neuroendocrinology. 2021;111:7. 10.1159/000509583. 10.1159/000509583 32570260

[107] De Francesco EM, Angelone T, Pasqua T, Pupo M, Cerra MC, Maggiolini M. GPER mediates cardiotropic effects in spontaneously hypertensive rat hearts. PLoS ONE. 2013;8:e69322. 23950890 10.1371/journal.pone.0069322 PMC3739764

[108] Lindsey SH, Cohen JA, Brosnihan KB, Gallagher PE, Chappell MC. Chronic treatment with the G protein-coupled receptor 30 agonist G-1 decreases blood pressure in ovariectomized mRen2.Lewis rats. Endocrinology. 2009;150:3753–8. 19372194 10.1210/en.2008-1664 PMC2717873

[109] da Silva JS, Sun X, Ahmad S, Wang H, Sudo RT, Varagic J, et al. G-Protein-Coupled Estrogen Receptor Agonist G1 Improves Diastolic Function and Attenuates Cardiac Renin-Angiotensin System Activation in Estrogen-Deficient Hypertensive Rats. J Cardiovasc Pharmacol. 2019;74:443–52. 31361702 10.1097/FJC.0000000000000721

[110] Dinh QN, Vinh A, Kim HA, Saini N, Broughton BRS, Chrissobolis S, et al. Aldosterone-induced hypertension is sex-dependent, mediated by T cells and sensitive to GPER activation. Cardiovasc Res. 2021;117:960–70. 32215568 10.1093/cvr/cvaa075

[111] Lindsey SH, da Silva AS, Silva MS, Chappell MC. Reduced vasorelaxation to estradiol and G-1 in aged female and adult male rats is associated with GPR30 downregulation. Am J Physiol. 2013;305:E113–8. 10.1152/ajpendo.00649.2012 PMC3725569 23673155

[112] Feldman RD, Gros R, Ding Q, Hussain Y, Ban MR, McIntyre AD, et al. A common hypofunctional genetic variant of GPER is associated with increased blood pressure in women. Br J Clin Pharmacol. 2014;78:1441–52. 25039431 10.1111/bcp.12471 PMC4256633

